# Susceptible genes and disease mechanisms identified in frontotemporal dementia and frontotemporal dementia with Amyotrophic Lateral Sclerosis by DNA-methylation and GWAS

**DOI:** 10.1038/s41598-017-09320-z

**Published:** 2017-08-21

**Authors:** E. Taskesen, A. Mishra, S. van der Sluis, R. Ferrari, International FTD-Genomics Consortium, D. G. Hernandez, M. A. Nalls, J. D. Rohrer, A. Ramasamy, J. B. J. Kwok, C. Dobson-Stone, P. R. Schofield, G. M. Halliday, J. R. Hodges, O. Piguet, L. Bartley, E. Thompson, E. Haan, I. Hernández, A. Ruiz, M. Boada, B. Borroni, A. Padovani, C. Cruchaga, N. J. Cairns, L. Benussi, G. Binetti, R. Ghidoni, G. Forloni, D. Albani, D. Galimberti, C. Fenoglio, M. Serpente, E. Scarpini, J. Clarimón, A. Lleó, R. Blesa, M. Landqvist Waldö, K. Nilsson, C. Nilsson, I. R. A. Mackenzie, G.-Y. R. Hsiung, D. M. A. Mann, J. Grafman, C. M. Morris, J. Attems, T. D. Griffiths, I. G. McKeith, A. J. Thomas, P. Pietrini, E. D. Huey, E. M. Wassermann, A. Baborie, E. Jaros, M. C. Tierney, P. Pastor, C. Razquin, S. Ortega-Cubero, E. Alonso, R. Perneczky, J. Diehl-Schmid, P. Alexopoulos, A. Kurz, I. Rainero, E. Rubino, L. Pinessi, E. Rogaeva, P. St George-Hyslop, G. Rossi, F. Tagliavini, G. Giaccone, J. B. Rowe, J. C. M. Schlachetzki, J. Uphill, J. Collinge, S. Mead, A. Danek, V. M. Van Deerlin, M. Grossman, J. Q. Trojanowski, J. van der Zee, C. Van Broeckhoven, S. F. Cappa, I. Leber, D. Hannequin, V. Golfier, M. Vercelletto, A. Brice, B. Nacmias, S. Sorbi, S. Bagnoli, I. Piaceri, J. E. Nielsen, L. E. Hjermind, M. Riemenschneider, M. Mayhaus, B. Ibach, G. Gasparoni, S. Pichler, W. Gu, M. N. Rossor, N. C. Fox, J. D. Warren, M. G. Spillantini, H. R. Morris, P. Rizzu, P. Heutink, J. S. Snowden, S. Rollinson, A. Richardson, A. Gerhard, A. C. Bruni, R. Maletta, F. Frangipane, C. Cupidi, L. Bernardi, M. Anfossi, M. Gallo, M. E. Conidi, N. Smirne, R. Rademakers, M. Baker, D. W. Dickson, N. R. Graff-Radford, R. C. Petersen, D. Knopman, K. A. Josephs, B. F. Boeve, J. E. Parisi, W. W. Seeley, B. L. Miller, A. M. Karydas, H. Rosen, J. C. van Swieten, E. G. P. Dopper, H. Seelaar, P. Scheltens, G. Logroscino, R. Capozzo, V. Novelli, A. A Puca, M. Franceschi, A. Postiglione, G. Milan, P. Sorrentino, M. Kristiansen, H.-H. Chiang, C. Graff, F. Pasquier, A. Rollin, V. Deramecourt, T. Lebouvier, D. Kapogiannis, L. Ferrucci, S. Pickering-Brown, A. B. Singleton, J. Hardy, P. Momeni, J. H. Veldink, M. A. van Es, A. B. Smit, D. Posthuma, Y. Pijnenburg

**Affiliations:** 1 VU University Amsterdam, Center for Neurogenomics and Cognitive Research, Complex Trait Genetics (CTG), Amsterdam Neuroscience, Amsterdam, The Netherlands; 2 VU University Medical Center (VUMC), Alzheimer Center, Amsterdam Neuroscience, Amsterdam, The Netherlands; 3 UCL London, Institute of Neurology, Department of Molecular Neuroscience, London, UK; 4 Department of Neurology, Brain Center Rudolf Magnus, University Medical Center Utrecht, Utrecht, The Netherlands; 5 VU University Amsterdam, Center for Neurogenomics and Cognitive Research, Department of Molecular and Cellular Neurobiology (MCN), Amsterdam Neuroscience, Amsterdam, The Netherlands; 6 Laboratory of Neurogenetics, National Institute on Aging, National Institutes of Health, Building 35, Room 1A215, 35 Convent Drive, Bethesda, MD 20892 USA; 7 Dementia Research Centre, Department of Neurodegenerative Disease, UCL Institute of Neurology, London, UK; 8 Department of Medical and Molecular Genetics, King’s College London Tower Wing, Guy’s Hospital, London, SE1 9RT UK; 9 Neuroscience Research Australia, Sydney, NSW 2031 Australia; 10 South Australian Clinical Genetics Service, SA Pathology at Women’s and Children’s Hospital, North Adelaide, SA 5006 Australia; 11 Research Center and Memory Clinic of Fundació ACE, Institut Català de Neurociències Aplicades, Barcelona, Spain; 12 Barbara Borroni (Neurology Clinic, University of Brescia, Brescia, Italy), Alessandro Padovani (Neurology Clinic, University of Brescia, Brescia, Italy; 13 Department of Psychiatry, Washington University, St. Louis, MO USA; 14 Department of Pathology and Immunology, Washington University, St. Louis, MO USA; 15 Molecular Markers Laboratory, IRCCS Istituto Centro San Giovanni di Dio Fatebenefratelli, Brescia, Italy; 16 MAC Memory Clinic, IRCCS Istituto Centro San Giovanni di Dio Fatebenefratelli, Brescia, Italy; 17 Biology of Neurodegenerative Disorders, IRCCS Istituto di Ricerche Farmacologiche, “Mario Negri”, Milano, Italy; 18 University of Milan, Milan, Italy; Fondazione Cà Granda, IRCCS Ospedale Maggiore Policlinico, via F. Sforza 35, 20122 Milan, Italy; 19 Memory Unit, Neurology Department and Sant Pau Biomedical Research Institute, Hospital de la Santa Creu i Sant Pau, Universitat Autònoma de Barcelona, Barcelona, Spain; 20 Unit of Geriatric Psychiatry, Department of Clinical Sciences, Lund University, Lund, Sweden; 21 Clinical Memory Research Unit, Department of Clinical Sciences, Lund University, Lund, Sweden; 22 Department of Pathology and Laboratory Medicine, University of British Columbia, Vancouver, Canada; 23 Division of Neurology, University of British Columbia, Vancouver, Canada; 24 Institute of Brain, Behaviour and Mental Health, University of Manchester, Salford Royal Hospital, Stott Lane, Salford, M6 8HD UK; 25 Department of Psychology, Weinberg College of Arts and Sciences, Northwestern University, Chicago, USA; 26 Newcastle University, Institute of Neuroscience and Institute for Ageing, Campus for Ageing and Vitality, NE4 5PL Newcastle upon Tyne, UK; 27 IMT School for Advanced Studies, Lucca, Lucca, Italy; 28 Taub Institute, Departments of Psychiatry and Neurology, Columbia University, 630 West 168th Street, New York, NY 10032 USA; 29 Behavioral Neurology Unit, National Insititute of Neurological Disorders and Stroke, National Insititutes of Health, 10 CENTER DR MSC 1440, Bethesda, MD 20892-1440 USA; 30 Department of Laboratory Medicine & Pathology, Walter Mackenzie Health Sciences Centre, 8440 - 112 St, University of Alberta Edmonton, Alberta, T6G 2B7 Canada; 31 Department of Neurology, Clínica Universidad de Navarra, University of Navarra School of Medicine, Pamplona, Spain; 32 Neuroepidemiology and Ageing Research Unit, School of Public Health, Faculty of Medicine, The Imperial College of Science, Technology and Medicine, London, W6 8RP UK; 33 Department of Psychiatry and Psychotherapy, Technische Universität München, Munich, 81675 Germany; 34 Neurology I, Department of Neuroscience, University of Torino, Italy, A.O. Città della Salute e della Scienza di Torino, Torino, Italy; 35 Tanz Centre for Research in Neurodegenerative Diseases, University of Toronto 60 Leonard Street, Toronto, Ontario, Canada M5T 2S8 USA; 36 Division of Neurology V and Neuropathology, Fondazione IRCCS Istituto Neurologico Carlo Besta, 20133 Milano, Italy; 37 Cambridge University Department of Clinical Neurosciences, Cambridge, CB2 0SZ UK; 38 University of California San Diego, Department of Cellular & Molecular Medicine, 9500 Gilman Drive, La Jolla, CA 92093 USA; 39 MRC Prion Unit, Department of Neurodegenerative Disease, UCL Institute of Neurology, Queen Square House, WC1N 3BG Queen Square, London UK; 40 Neurologische Klinik und Poliklinik, Ludwig-Maximilians-Universität, German Center for Neurodegenerative Diseases (DZNE), Munich, Germany; 41 University of Pennsylvania Perelman School of Medicine, Department of Pathology and Laboratory Medicine, Philadelphia, PA USA; 42 Neurodegenerative Brain Diseases group, VIB-UAntwerp Center of Molecular Neurology, Antwerp, Belgium; Laboratory of Neurogenetics, Institute Born-Bunge, University of Antwerp, Antwerp, Belgium; 43 Neurorehabilitation Unit, Dept. Of Clinical Neuroscience, Vita-Salute University and San Raffaele Scientific Institute, Milan, Italy; 44 Inserm, UMR_S975, CRICM; UPMC Univ Paris 06, UMR_S975; CNRS UMR 7225, F-75013, Paris, France; AP-HP, Hôpital de la Salpêtrière, Département de neurologie-centre de références des démences rares, F-75013 Paris, France; 45 Service de Neurologie, Inserm U1079, CNR-MAJ, Rouen University Hospital, Rouen, France; 46 Department of Neurosciences, Psychology, Drug Research and Child Health (NEUROFARBA) University of Florence, Florence, Italy; 47 Danish Dementia Research Centre, Neurogenetics Clinic, Department of Neurology, Rigshospitalet, Copenhagen University Hospital, Copenhagen, Denmark; 48 Saarland University, Laboratory for Neurogenetics, Kirrberger Str.1, Bld.90, 66421 Homburg/Saar, Germany; 49 University Regensburg, Department of Psychiatry, Psychotherapy and Psychosomatics, Universitätsstr. 84, 93053 Regensburg, Germany; 50 University of Cambridge, Department of Clinical Neurosciences, John Van Geest Brain Repair Centre, Forvie Site, Robinson way, Cambridge, CB2 0PY UK; 51 German Center for Neurodegenerative Diseases-Tübingen, Otfried Muellerstrasse 23, Tuebingen, 72076 Germany; 52 Regional Neurogenetic Centre, ASPCZ, Lamezia Terme, Italy; 53 Department of Neuroscience, Mayo Clinic Jacksonville, 4500 San Pablo Road, Jacksonville, FL 32224 USA; 54 Department of Neurology, Mayo Clinic Rochester, 2001st street SW, Rochester, MN 5905 USA; 55 William W Seeley (Department of Neurology, Box 1207, University of California, San Francisco, CA 94143 USA; 56 Memory and Aging Center, Department of Neurology, University of California, San Francisco, CA 94158 USA; 57 Department of Neurology, Erasmus Medical Centre, Rotterdam, The Netherlands; 58 Alzheimer Centre and department of neurology, VU University medical centre, Amsterdam, The Netherlands; 59 Department of Basic Medical Sciences, Neurosciences and Sense Organs of the “Aldo Moro” University of Bari, Bari, Italy; 60 Medical Genetics Unit, Fondazione Policlinico Universitario A. Gemelli, Rome, Italy; 61 Cardiovascular Research Unit, IRCCS Multimedica, Milan, Italy; 62 Neurology Dept, IRCCS Multimedica, Milan, Italy; 63 Department of Clinical Medicine and Surgery, University of Naples Federico II, Naples, Italy; 64 UCL Genomics, Institute of Child Health (ICH), UCL, London, UK; 65 Karolinska Institutet, Dept NVS, Alzheimer Research Center, Novum, SE-141 57 Stockholm, Sweden; 66 Univ Lille, Inserm 1171, DISTALZ, CHU 59000 Lille, France; 67 National Institute on Aging (NIA/NIH), 3001 S. Hanover St, NM 531, Baltimore, MD 21230 USA; 68 Clinical Research Branch, National Institute on Aging, Baltimore, MD USA; 69 Laboratory of Neurogenetics, Department of Internal Medicine, Texas Tech University Health Science Center, 4th street, Lubbock, Texas 79430 USA

## Abstract

Frontotemporal dementia (FTD) is a neurodegenerative disorder predominantly affecting the frontal and temporal lobes. Genome-wide association studies (GWAS) on FTD identified only a few risk loci. One of the possible explanations is that FTD is clinically, pathologically, and genetically heterogeneous. An important open question is to what extent epigenetic factors contribute to FTD and whether these factors vary between FTD clinical subgroup. We compared the DNA-methylation levels of FTD cases (n = 128), and of FTD cases with Amyotrophic Lateral Sclerosis (FTD-ALS; n = 7) to those of unaffected controls (n = 193), which resulted in 14 and 224 candidate genes, respectively. Cluster analysis revealed significant class separation of FTD-ALS from controls. We could further specify genes with increased susceptibility for abnormal gene-transcript behavior by jointly analyzing DNA-methylation levels with the presence of mutations in a GWAS FTD-cohort. For FTD-ALS, this resulted in 9 potential candidate genes, whereas for FTD we detected 1 candidate gene (*ELP2*). Independent validation-sets confirmed the genes *DLG1, METTL7A, KIAA1147, IGHMBP2, PCNX, UBTD2, WDR35*, and *ELP2/SLC39A6* among others. We could furthermore demonstrate that genes harboring mutations and/or displaying differential DNA-methylation, are involved in common pathways, and may therefore be critical for neurodegeneration in both FTD and FTD-ALS.

## Introduction

Frontotemporal dementia (FTD) is a rare neurodegenerative disorder with estimated point prevalence of approximately 15–30 per 100,000 individuals in the age group 49–69 years^1,2^. FTD shows progressive deterioration of behavior and cognition, and gives raise to various clinical subtypes, such as the behavioral variant (bvFTD), characterized by e.g. changes in personality, and semantic dementia and progressive non-fluent aphasia subtypes, characterized by language-associated variants. FTD also co-occurs with Amyotrophic Lateral Sclerosis (FTD-ALS), approximately seen in 15% of all FTD cases^3^, which forms a reason why FTD and ALS may be considered as a disease continuum. The clinical symptoms of FTD are related to selective neurodegeneration of the frontal brain regions, anterior temporal brain regions, often in conjunction with the degeneration of subcortical brain regions. The two most prevalent pathologies within the clinical spectrum of FTD are frontotemporal lobar degeneration (FTLD) with TAR DNA- binding protein 43 inclusions (FTLD-TDP), and FTLD with tau positive inclusions (FTLD-Tau).

Variation in microtubule-associated protein tau (*MAPT*), progranulin (*GRN*) and Chromosome 9 open reading frame 72 (*C9orf72*) is the most frequent genetic cause of familial FTD, together representing 10–27% of all FTD cases^4,5^. It is yet to be elucidated to what extent genetic variation accounts for sporadic FTD^6^. Genome-wide association studies (GWAS) have become a standard approach to identify common genetic risk variants for complex diseases. In the case of FTD, the largest GWAS to date engaged data of 3,526 patients^4^, and revealed 2 loci that included genes involved in immune system processes, and genes involved with lysosomal and autophagy pathways. However, the two detected loci can only partly explain the causation of FTD, which raises the question to what extent other molecular factors contribute to the pathogenesis in FTD. The clinical, pathological, and genetic heterogeneity of FTD might hamper identification of genes for FTD. Moreover, it has been shown that toxicity by specific mutations (i.e., *C9orf72*) might depend on the expression level of a gene^7^. Because cytosine DNA-methylation can also regulate the expression of genes, various studies performed single promoter analysis for *C9orf72*^8–10^, and *GRN*^11,12^, and showed the importance of DNA promoter hypermethylation in less severe clinical outcome. However, whole genome epigenetic involvement in FTD, to identify potential associations with pathogenesis, such as degeneration of the frontal and temporal lobes, has as yet to be elucidated^13^. The role of DNA-methylation is well established in the field of cancer^14^, where it showed promising clinical and preclinical results with the development of drugs targeting chromatin regulators^15^. For neurological diseases and dementia, the role of epigenetics is increasingly recognized^16–18^. Nevertheless, studying the role of epigenetics in brain disorders remains a challenging task as DNA-methylation is usually measured in blood instead of brain tissue. For FTD and PSP (Progressive Supranuclear Palsy), a large genome-wide epigenetic study (DNA-methylation profiles) has been conducted, demonstrating a mediating role for methylation in PSP^19^. However, the exact role of genome-wide DNA-methylation for patients with FTD with or without concomitant Amyotrophic Lateral Sclerosis (FTD-ALS) has not been established.

Here, we study genome-wide DNA-methylation profiles of the FTD cases in the previous cohort (total n = 128, of which 7 cases are FTD-ALS), and separately for the clinical subtype FTD-ALS. We aimed to: (1) Explore separation of FTD clinical subtypes using the DNA-methylation profiles. (2) detect genetic variants and/or epigenetic changes that show associations with FTD and/or FTD-ALS, and (3) examine whether genetic and epigenetic risk factors for FTD and/or FTD-ALS converge into specific biological processes as these may indicate evidence for a role of epigenetics in neurodegeneration in FTD.

## Results

### The clinical subtype FTD-ALS showed significant class separation from controls using DNA-methylation profiles

**Figure 1:**
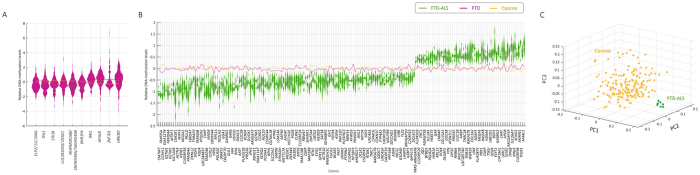
Differential methylated genes for FTD and FTD-ALS. Violin plot depicting the significant differential cytosine DNA-methylated genes are depicted for (**A**) all the 128 FTD cases, and (**B**) the FTD-ALS cases by comparison to control cases. Genes are sorted based on T-statistics. Genes with relative value lower than 0 are DNA hypomethylated and relative value higher than 0 are DNA hypermethylated. The green, purple and yellow lines depict the average value for respectively FTD-ALS, FTD and control cases. (**C**) Principal Component analysis on the differential DNA-methylated probes from FTD-ALS.

The comparison of all FTD cases (n = 128) versus controls (n = 193) revealed 10 significantly differential cytosine DNA-methylated probes (annotated with 14 unique genes) after multiple test correction for the 214,170 genes using Benjamin and Hochberg (*P*_BH_ < 0.05, Fig. 1A, Table S1, and Fig. S1 panel A,B). These include genes with brain and/or neurological function, such as Thiamin Pyrophosphokinase 1 (*TPK1*), which has been associated with psychomotor retardation^20^ and for which a missense/splice-site/frameshift mutation results in progressive neurological dysfunction. We also detected Serine Threonine Kinase 39 (*STK39*), which is involved in Parkinson Disease^21,22^, Retinoic Acid Induced 1 (*RAI1*), which is involved in the control of early neural differentiation), and Solute Carrier Family 39 (*SLC39A6*), which belongs to a subfamily of proteins that show structural characteristics of zinc transporters^23^ and which is associated with length of survival in esophageal squamous-cell carcinoma^24^, and overexpressed in Frontal cortex. Note that we will use *P*_BH_ in the manuscript as Benjamin and Hochberg corrected P-value. We next assessed whether the entire cohort of FTD cases had a unique methylation profile and grouped separately from controls by means of an unsupervised analysis. We performed hierarchical clustering for which the optimal tree cut-off was determined by the Davies-Bouldin index. No class separation between cases and controls was detected (Fig. S1C).

Comparison of the FTD-ALS cases (n = 7) to the controls resulted in 200 significant differential cytosine DNA-methylation probes (*P*_BH_ < 0.05, Fig. S1 panel A-B), annotated for 224 unique genes (Fig. 1B). Note that none of the 14 genes identified for FTD were found among the 224 genes for FTD-ALS. Moreover, 140 probes (mapped to 163 genes) showed relatively lower methylation levels compared to controls with average value below 0, indicating a DNA hypomethylation state. The remaining 60 probes (mapped to 63 genes) showed, with an average value above 0, relatively higher methylation levels then controls indicating a DNA hypermethylation state (Figs 1B, S2B). The unique DNA-methylation profiles were even more stressed by the Principal Component Analysis, and Davies-Bouldin index to determine the number of clusters, which resulted in an exclusive and significant grouping of the FTD-ALS cases (*P* = 5.23 × 10^−11^, Fig. 1C).

### Genes that are specific for brain tissue show significant overlap with the associated FTD-ALS DNA-methylated genes

**Figure 2:**
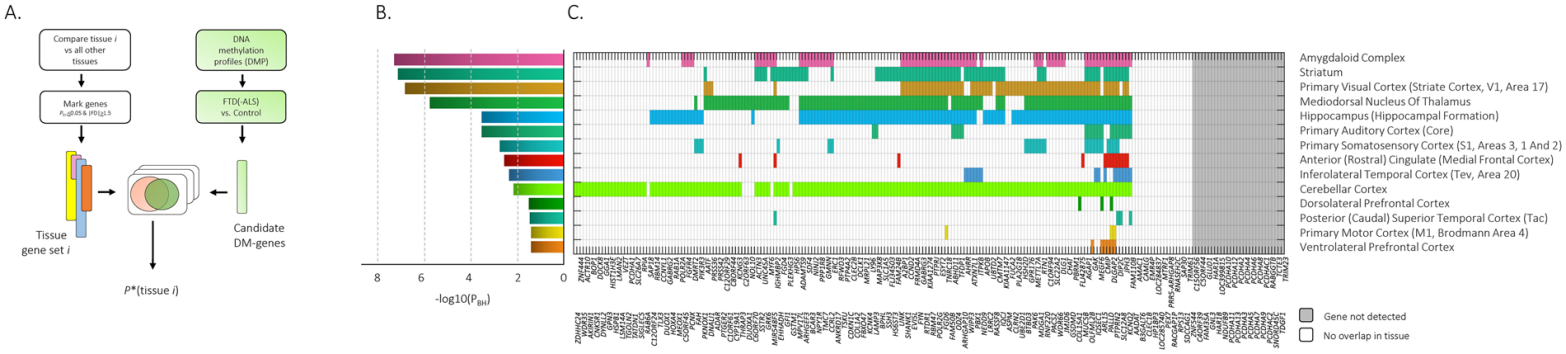
Tissue enrichment for FTD-ALS based on the brain tissue specific DNA-methylation profiles of BrainSpan. (**A**) The hypergeometric test is used to compute *P*-value for each tissue based on the following parameters; total number of genes (M), number of tissue specific genes (K), number of significant differential methylated genes (N), and the overlap of significant differential methylated genes and the genes in the tissue specific gene set (x). The final *P*-value (P*) is corrected for multiple testing using Benjamini and Hochberg, and used for tissue selection with *P*_BH_ < 0.05. (**B**) Enriched tissues in FTD-ALS sorted in -log10(*P*_BH_). (**C**) Genes are colored with the brain tissue specific color if overlap is seen with any of the significantly differential DNA-methylated genes in FTD-ALS.

To test whether the detected differential DNA-methylation genes for FTD (n = 14 genes) and, FTD-ALS (n = 224 genes) are associated with expression in brain, we utilized RNA sequencing data, containing 16,115 expression levels of genes, from the GTEx consortium on 1,641 samples and over 25 unique tissue types^25–27^ (more details can be found in method section: Tissue-type association). To determine tissue enrichment, we marked the genes that are specific for each tissue-type by comparing tissue-specific versus remaining samples, under the restriction that expression levels were significantly different with *P*_BH_ < 0.05 (corrected for 16,115 tests) using the Students T-test and with absolute Fold-difference of >1.5 (Fig. 2A). Genes associated with FTD and FTD-ALS were subsequently tested for significant enrichment for any of the tissue-specific-gene sets using the hypergeometric test.

No tissue-specific significant enrichment was seen for the 14 FTD associated differentially DNA-methylated genes after multiple test correction. For FTD-ALS cases, we did detect three significantly associated tissue types out of 25 tissues tested, namely Blood (*P*_BH_ = 0.01), Brain (*P*_BH_ = 0.02), and Liver (*P*_BH_ = 0.04) (Fig. S1A), based on the 224 differential DNA-methylated genes. More specific, across the brain regions we detected significant overrepresentation for Parietal Neocortex (*P*_BH_ = 9.41 × 10^−4^) and Primary Motor-Sensory Cortex (*P*_BH_ = 0.031). This indicates that methylation changes detected in peripheral blood of FTD-ALS cases could also be reflective of changes in other tissues, including the brain.

### DNA-methylation profiles of FTD-ALS patients reflect biological processes essential in Prefrontal, Primary Motor-Sensory Cortex, and Parietal Neocortex

Next, we addressed the question whether the hyper/hypo DNA-methylated genes in FTD, and separately in FTD-ALS, are significantly overrepresented among genes that are specific for any of the brain regions (instead of tissue types as demonstrated in the previous section). We tested for significant overrepresentation based on RNA-sequencing (525 samples across 26 brain regions), DNA-methylation (177 samples over 17 brain regions), and pre-defined gene sets (n = 22) from BrainSpan^28^ by using the procedure as outlined in Fig. 2A.

For the 224 associated genes in FTD-ALS, we detected significant overrepresentation with Parietal Neocortex (*P*_*BH*_ = 9.41 × 10^−4^) and Primary Motor-Sensory Cortex (*P*_*BH*_ = 0.031, Fig. S3B) using the RNA-sequencing data, after correcting for the 26 performed tests. Based on the DNA-methylation profiles, we detected significant overrepresentation in 14 specific brain regions (Fig. 2B), among which Primary Visual Cortex (*P*_BH_ = 2.05 × 10^−7^), Primary Motor Cortex (*P*_BH_ = 0.0441), Dorsolateral Prefrontal Cortex (*P*_BH_ = 0.0351), and Inferolateral Temporal Cortex (*P*_BH_ = 0.0051), after correcting for the 17 performed tests. Finally, for the pre-defined gene sets we detected borderline significance for the Medial Prefrontal Cortex tissue (*P*_*BH*_ = 0.05). In general, we observed that the majority of DNA-methylated genes in FTD-ALS (176/224, Fig. 2C) overlaps with the genes that are significantly differentially expressed in any of the 14 brain regions. For FTD we detected no significant overrepresentation of the 14 genes among any of the brain specific regions (*P*_*BH*_ < 0.05).

### Candidate genes from GWAS revealed by joint analysis with the DNA-methylation profiles

We hypothesized that genes that contain potential risk SNPs and have a differential DNA-methylation profile, may have increased susceptibility for differences in gene-transcript levels, and may therefore be implicated in the disease development. To test this hypothesis, we utilized GWAS summary statistics for FTD, and separately for FTD-ALS^4^, and extracted all SNPs with unadjusted *P* < 0.05. Note that the corrected *P*-value threshold for GWAS does only yield in few genes but we hypothesized that multiple but relatively smaller effects can have impact on the functional level.

For FTD-ALS this yielded 5,535 SNPs, annotated to 4,147 unique genes using ANNOVAR^29^. First, we overlaid the 4,147 genes with the 224 genes *as per* the FTD-ALS DNA-methylation markers and detected a significant overlap based on the hypergeometric test (53 genes, *P* = 0.0005, Table S2, using as background the total number of unique HG19 genes). This indicates that in FTD-ALS, non-random genes were detected with both risk SNPs and differences in DNA-methylation levels. To further refine the potential candidate genes, we removed intronic, intergenic and synonymous SNPs and incorporated the CADD score to determine the deleteriousness. This filtering step yielded in 26 candidate genes for 30 SNPs (with CADD-score > 15) that are nonsynonymous or stopgain in exonic or splicing regions (Table 1). The 26 genes could be categorized into genes with DNA hypermethylation (n = 8) and hypomethylation (n = 18) status. None of the 30 SNPs occurred exactly in a DNA-methylation probe-region.

**Table 1:** Candidate list of genes that display abnormal DNA-methylation levels, and harbor risk-SNPs.

Genesymbol	Chr	Strand	GWAS SNPs					DNA-methylation probes				Network	Pathway	Validated by gene-expression
			Id	*P*	Location	CADD score	min(P) for gene	Id	*P*_BH_	Location	Tstat	Gene-degree	Overlap	
*DLG1*	chr3	−	rs74674649	6.01E-04	exonic	28.2	6.01E-04	cg12594803	0.0288	.	–7.04	6	Yes	Yes
*KIAA1147*	chr7	−	rs201876806	9.13E-04	exonic	23.4	9.13E-04	cg24662653	2.90E-03	Island	–6.51	2		Yes
*GGA1*	chr22	+	rs143909159	2.91E-03	exonic	34	2.91E-03	cg21268578	0.028	.	–7.16	21		
*IGHMBP2*	chr11	+	rs201692151	4.00E-03	exonic	22.8	4.00E-03	cg26065952	0.0231	N_Shore	–5.7	31		Yes
*ASPM*	chr1	−	rs150125249	5.78E-03	exonic	34	5.78E-03	cg11336294	0.0273	.	–5.59	.	Yes	
*PRSS36*	chr16	−	rs145749002	6.04E-03	exonic	33	6.04E-03	cg14301190	4.90E-03	Island	–6.68	1		
*CNKSR1*	chr1	+	rs144396219	8.32E-03	exonic	27.7	8.32E-03	cg09890400	0.0196	.	–8.83	11		Yes
*UBTD2*	chr5	−	rs17074452	9.54E-03	exonic	18.33	9.54E-03	ch.5.3268483F	4.90E-03	.	–5.84	12		Yes
*GGA1*	chr22	+	rs138525343	9.76E-03	exonic	26.6	2.91E-03	cg21268578	0.028	.	–7.16	21		
*IGHMBP2*	chr11	+	rs145226920	0.01001	exonic	35	4.00E-03	cg26065952	0.0231	N_Shore	–5.7	31		
*FAH*	chr15	+	rs144234072	0.01066	exonic	27.3	0.01066	cg06856840	0.0165	.	–6.95	2	Yes	
*NEDD9*	chr6	−	rs34044517	0.01629	exonic	23.6	5.85E-03	cg05917225	9.20E-03	.	–5.79	2		
*BPHL*	chr6	+	rs2231365	0.01671	splicing	24.1	0.01671	cg22799902	6.20E-03	Island	–6.43	2		
*ACTN3*	chr11	+	rs201576110	0.02133	exonic	32	0.02133	cg25117505	6.39E-06	Island	–8.39	.		
*WDR66*	chr12	+	rs77422261	0.02336	exonic	29.7	0.02336	cg21016266	0.0133	Island	–4.89	3		
*IQSEC. 1*	chr3	−	rs144790333	0.02763	exonic	23	0.02763	cg02559896	6.70E-03	Island	–5.14	16	Yes	
*DLG1*	chr3	−	rs141544348	0.03024	exonic	35	6.01E-04	cg12594803	0.0288	.	–7.04	6		
*DUOX1*	chr15	+	rs143304688	0.03047	exonic	44	0.03047	cg10496082	2.00E-04	Island	–6.61	4	Yes	
*PCDHA3*	chr5	+	rs146951816	0.0354	exonic	15.16	0.0354	cg02357321	0.0335	N_Shore	–6.43	.		
*C6orf70*	chr6	+	rs140632188	0.04056	exonic	23	0.04056	cg22807378	0.0121	Island	–7.15	1		
*CLRN2*	chr4	+	rs201124485	0.04711	exonic	20.6	0.04711	cg16760587	1.10E-03	S_Shelf	–6.06	.		
*COL15A1*	chr9	+	rs199906142	3.62E-03	exonic	23.1	3.62E-03	cg18115656	0.0366	Island	5.37	0	Yes	
*TNRC18*	chr7	−	rs112785272	6.27E-03	exonic	19.2	6.27E-03	cg10546562	0.0101	N_Shore	5.91	28	Yes	
*SLC26A7*	chr8	+	rs200788056	9.04E-03	exonic	23.3	9.04E-03	cg25481252	2.60E-03	.	7.38	.		
*PCNX*	chr14	+	rs200261097	0.01309	exonic	32	0.01309	cg10066683	0.0422	.	5.34	31	Yes	Yes
*WDR35*	chr2	−	rs148436608	0.02293	exonic	16.34	0.02293	cg13734338	0.028	.	6	0		Yes
*MEGF6*	chr1	−	rs61910697	0.03109	exonic	19.86	0.03109	cg04391135	3.50E-03	Island	6.21	0		
*PRR5-ARHGAP8*	chr22	+	rs55849456	0.03933	exonic	24.6	0.03933	cg06647930	0.0442	S_Shelf	4.26	.		Yes
*PRR5-ARHGAP8*	chr22	+	rs16992915	0.04662	exonic	19.38	0.03933	cg06647930	4.42E-02	S_Shelf	4.26	.		Yes
*ESYT2*	chr7	−	rs2305475	0.04728	exonic	23	0.04728	cg19584649	9.36E-05	.	6.66	15		Yes

**Figure 3:**
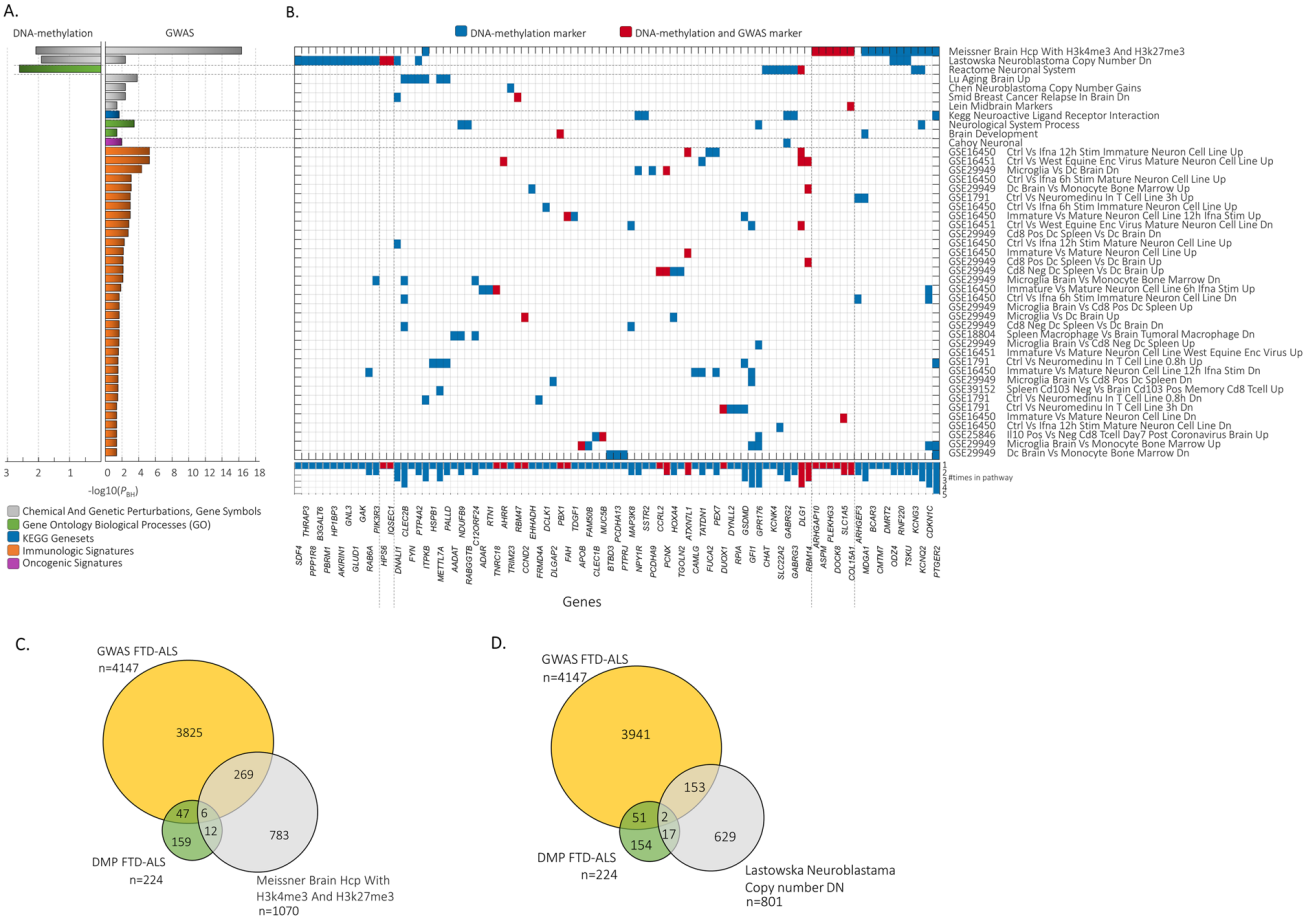
Enriched pathways for FTD-ALS. (**A**) Significantly enriched pathways using the DMP and GWAS associated genes of FTD-ALS. (**B**) Blue colored squares depict overlap of genes between FTD-ALS markers and pathway associated genes, whereas a red colored square also depicts overlap with the GWAS associated genes. (**C**) Venn diagram depicts the overlap between FTD-ALS genes from DMP (n = 224), GWAS (n = 4147), and the genes in gene set Meissner Brain Hcp with H3k4me3 and H3k27me3 (n = 1070), and (**D**) Lastowska Neuroblastama Copy number DN (n = 801).

**Figure 4:**
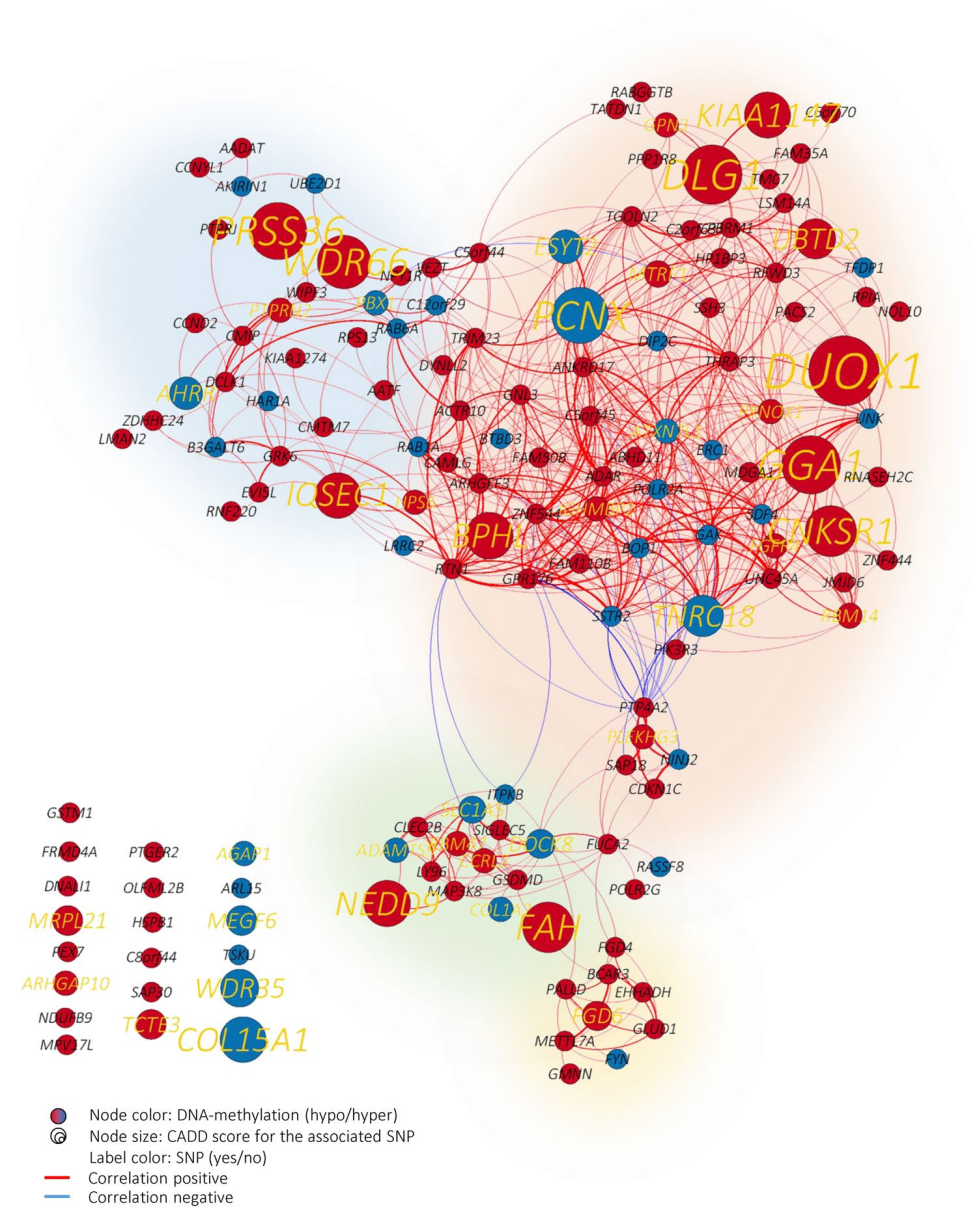
Co-expression network for FTD-ALS group. Continuous gene expression data from GTEx (n = 313 samples over 13 brain regions) was used to build a co-expression network using the genes that are marked being significantly differential DNA-methylated in FTD-ALS group, and with significant pairwise correlation (|r| > 0.6 and *P* < 0.001). Node color depicts DNA-methylation status of the gene; red color depicts DNA hypomethylation (T-statistics < 0), and blue color depicts DNA hypermethylation (T-statistics > 0). Node size and text label depicts CADD score for the associated SNP (larger node size depicts a relatively more deleterious variant, and genes without a SNP have equal small node size). Yellow colored text labels depict a SNP that associated with the gene from GWAS FTD-ALS, whereas a black color depicts no SNP. Edges with positive correlation are indicated in red, whereas negative correlations are indicated in blue. Thickness of edges is based on the absolute correlation measure, which varies between 0.6 and 1.

Detection of 26 candidate genes for 30 SNPs for FTD-ALS. Genes are grouped in DNA hypomethylated (DMP T-statistics < 0) and hypermethylated genes (DMP T-statistics > 0) followed by *P*-value significance of GWAS. Chr: Chromosome. GWAS P: *P*-value significance for the phenotype association. GWAS CADD score: quantifies the deleteriousness of the SNP in the gene (the higher the worse). GWAS min(P) for gene: Minimum *P*-value significance for the phenotype association without excluding intronic, and intergenic SNPs. DMP *P*_BY_: P-value for the DNA-methylation difference between FTD-ALS vs Control cases after multiple test correction using Benjamini and Hochberg. DMP T-stat: T-statistics. Network Gene-degree: the number of edges the gene contains in the co-expression network (Fig. 4). Pathway overlap: [Yes], if the gene overlaps with any of the pathways derived from DMP or GWAS (Fig. 3A and B). Validated by gene-expression: [Yes], if the gene showed significant differential expression in any of the validation data sets.

The most significant SNP association, detected in gene *DLG1*, is exonic located (rs74674649, *P* = 6.0 × 10^−4^), and the promoter region of the gene also harbors a significant hypomethylation status (*P* = 0.0288). This gene is described as being exclusively located in the postsynaptic density of neurons, and is crucially involved in anchoring postsynaptic membrane proteins.

A similar approach was performed for all FTD cases but here we extracted SNPs with unadjusted *P* < 0.05 using the summery statistics of the FTD-GWAS (instead of FTD-ALS). Positional mapping of SNPs using ANNOVAR revealed 3,662 genes. We detected 4 overlapping genes (*P* = 0.0553, Table S3) between the 14 DMP genes and 3,662 GWAS genes. One out of the four genes; *ELP2*, contained a SNP (rs16967474, *P* = 0.0322) that was exonic located, being nonsynonymous, and with CADD-score of 25.3. Interestingly the *ELP2* gene was recently found implicated in neurodevelopmental disabilities^30^. To summarize, we here isolated potentially functionally relevant genes for FTD, particularly for the FTD-ALS subtype, based on the combination of both genetic and epigenetic profiles.

### Biological processes are affected by both genetic and epigenetic aberrations

To assess whether biological mechanisms are affected in FTD-ALS, either due to differences in DNA-methylation levels (n = 224 genes) or due to genetic architecture (n = 4,147 genes), we performed a pathway analysis on the 224 genes, and separately 4,147 genes. We next analyzed the overlap of pathways. Note that we did not detect significant enrichment of pathways for the 14 unique markers in FTD by means of the hypergeometric test.

Pathway analysis was performed by using gene sets with a described function in brain and/or neurological development, and were derived from the molecular signature database (MsigDB v5.1^31^, see methods section for more details, such as the number of pathways that were tested). The 224 DMP genes for FTD-ALS revealed three significantly enriched pathways (*P*_BH_ < 0.05, Fig. 3A), namely: Reactome Neuronal System (*P*_BH_ = 0.005), Lastowska Neuroblastoma Copy Number DN (*P*_BH_ = 0.0256), and Meissner brain HCP with H3K4me3/H3K27me3 (*P*_BH_ = 0.0182). Separately, we performed a pathway analysis for the 4,147 unique genes derived from the FTD-ALS GWAS, which resulted in 44 enriched pathways (*P*_BH_ < 0.05, Fig. 3A). Two of three pathways overlapped, i.e., Meissner brain HCP with H3K4me3/H3K27me3 (*P*_BH_ = 6.82 × 10^−11^), and Lastowska Neuroblastoma Copy Number DN (*P*_BH_ = 8.45 × 10^−4^). The histone modification H3K4me3/H3K27me3 gene set was previously implicated in various neurological phenotypes and psychiatric disorders^32^, whereas the neuroblastoma pathway points to genes with copy-number losses in primary neuroblastoma tumors for which neuroblastoma cell lines were also used as a model-system for FTD^33,34^.

Interestingly, the two common pathways showed different overlapping genes (Fig. 3B), indicating that different genes are implicated from the genetic and epigenetic perspective but are located in the same pathway. As an example, the histone modification H3K4me3 gene set contains 1070 genes with only a joint overlap of six genes between the genetic and epigenetic markers (Fig. 3C). Similarly, the Lastowska Neuroblastoma Copy Number DN gene set contains 801 genes with a joint overlap of two genes (Fig. 3D).

### DNA-methylated genes involved in FTD-ALS are highly co-expressed in normal brain function

To analyze the mediating role of DNA-methylation on the signaling cascade in FTD-ALS, we constructed a co-expression network (pairwise Spearman correlations) between the continuous mRNA expression levels using data from the GTEx consortium (see methods section for more details). The co-expression network contained 150 genes (out of the 224 genes) with minimum correlation of |r| > 0.6 and significant pairwise interactions *P* < 0.001 (Fig. 4).

In the co-expression network topology, we overlaid: (1) DNA-methylation status of FTD-ALS cases (node color); (2) The detected SNPs from GWAS FTD-ALS cases (marked with yellow colored gene label), and; (3) The associated CADD-score (node size). To get a notion of the functional importance of a gene, we used the gene-degree in the co-expression network (number of edges the gene contains) as higher regulators may have more co-expressed genes. We used gene-degree in the co-expression network to further prioritize the candidate gene-list (Table 1, Fig. 4). We detected that Immunoglobulin Mu Binding Protein 2 (*IGHMBP2)* was one of the genes with highest degree (31) that also contained a deleterious stop-gain mutation (CADD score: 22.8). Interestingly, this gene is associated with the disease distal hereditary motor neuropathy type 6, where motor neurons degenerate selectively in the anterior horn of the spinal cord. The full list of gene-degrees is listed in Table S4.

### DNA-methylation levels for GRN, MAPT, and C9orf72

Besides analyzing the methylation profiles from a genome-wide perspective, we also analyzed separately the probes associated with the three known genetic markers of FTD, i.e., *GRN, MAPT*, and *C9orf72*.

The promoter of GRN has previously been demonstrated to be hypermethylated^11^. In our data set, 12 GRN probes were available for which one probe (cg17101358, located at 5′UTR/1stExon) resulted in borderline significant differences in DNA-methylation levels (P_BH_ = 0.059) in FTD, (compared to the control group with Student T-test). No significant difference in DNA-methylation levels were detected for the FTD-ALS group. The gene MAPT contained one probe but without significant differences in DNA-methylation levels for both FTD, and FTD-ALS cases, which is in line with current literature^35,36^. Analysis of the four C9orf72 probes (5′UTR, TSS200, and two in TSS1500) did also not result in significant differences in DNA-methylation for FTD, nor FTD-ALS cases. Note that C9orf72 has previously been identified with DNA hypermethylation in the promoter region when performing a single-gene promoter analysis^37^.

### Validation by meta-analysis of gene transcript levels

We sought replication to examine the validity of the detected genes that reached genome-wide significance in the primary analyses. Since there are no independent DNA-methylation profiles for FTD or FTD-ALS, we used gene transcript levels of samples with FTD, and separately Amyotrophic Lateral Sclerosis cases (ALS), which is similar to ALS in FTD-ALS. The mediating role of DNA-methylation on the transcript level is well established, and therefore we hypothesized that similar affected genes should be evident from our study. We included four independent studies from Gene Expression Omnibus (GEO) that we considered the most suitable for validation. We analyzed these data sets in a meta-analysis (see materials and methods), where we ranked the DNA-methylated genes, implicated in FTD or FTD-ALS, based on the overlap with the significantly differential expressed genes across the seven validation data sets.

To determine the significantly differential expressed genes across the validation data sets, we performed an unbiased test by comparing the gene expression levels of cases versus controls using Limma. Note that we multiple test corrected for the number of probes that were present per study as described in Materials and methods section. All validation data sets, except one (#4), resulted in significantly differential expressed genes (*P*_*BH*_ < 0.05, Tables S5 and S6).

**Figure 5:**
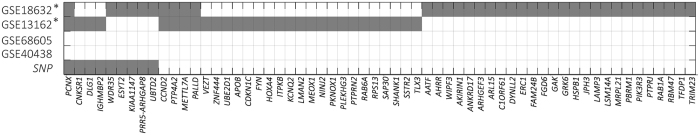
Validated genes by independents gene expression data sets. Significantly differential expressed genes across six independent studies overlaid with the aberrant DNA-methylated genes in FTD-ALS. Grey squares depict overlap of genes between FTD-ALS markers and one or multiple validation data set. Genes that could not be validated were removed from the plot. The SNP row depicts genes that were also discovered with a deleterious SNP.

For FTD-ALS, 60 out of 224 genes could be validated in total (Fig. 5, Table S5) from which 5 genes were seen across two validation sets; *CCND2, PCNX, PTP4A2, METTL7A*, and *PALLD*. To further specify potential candidate genes that are implicated in FTD-ALS, we only included genes with aberrant DNA-methylation and deleterious SNPs, and detected 9 genes (Table 1, and Fig. 5). For FTD we detected one gene, namely *ELP2/SLC39A6* (Table S1). Besides the validation of single genes, we also emphasized the relevance of our DNA-methylated gene set of FTD-ALS by the detection of significant overrepresentation of genes across two validation data sets (#1, and #2, Fisher exact test, *P* < 0.05, Table S6). No significant results were seen for FTD.

## Discussion

In this study, we investigated the DNA-methylation profiles (DMPs) of cases with FTD to detect genes affected by epigenetic biological mechanisms that may play a role in neurodegeneration. The first aim in this study was to explore the separation of FTD clinical subtypes using the DNA-methylation profiles for which we could demonstrate a clear separation of the FTD-ALS subtype. The second aim was to detect genetic variants and/or epigenetic changes that show associations with FTD and/or FTD-ALS. Ideally the candidate genes should be validated with bisulfite pyrosequencing or using an independent DNA-methylation cohort of FTD cases but such a data set does not exist in the public domain. We aimed to validate our results by using multiple independent gene expression data sets. The validates genes have thus increased susceptibility for abnormal gene-transcript behavior, harbor risk-SNPs, and display abnormal DNA-methylation levels, and many are annotated with function in brain and/or neurodevelopment.

Depending on the follow-up steps, the gene-list can be further narrowed by specific ordering, e.g., based on SNP association, DNA-methylation status, degree of co-expression, or even by its role in specific pathways. As an example, synapse-associated gene *DLG1* contains the most significant SNP association followed by *KIAA1147* which is suggested to have a role in neurogenesis and neuronal recovery and/or restructuring in the hippocampus following transient cerebral ischemia^38^. For the validated genes with hypomethylation status, we identified gene *KIAA1147*, and gene *IGHMBP2* among others. The latter gene is described with distal hereditary motor neuronopathy type 6, which selectively degenerates motor neurons in the anterior horn of the spinal cord, and reported with a role in development of adult human brain, and motor neurons^39^. Prioritization based on the co-expression networks placed gene *IGHMBP2*, and *PCNX* as the top genes. Notably, genes without a deleterious SNP can also be of interest and ordered based on degree of co-expression. An example is gene *GPR176* (degree = 32) which is involved in responses to hormones, growth factors, and neurotransmitters^40^, whereas gene *ATXN7L1* (degree = 32) showed functional relation to brain based on the Human Integrated Protein Expression Database (HIPED). Another gene of interest with DNA hypermethylation status is *COL15A1*, which is previously reported with downregulated expression levels in iPSC-derived ALS motor neurons^41,42^. Our results are in line with these findings as the hypermethylation in the promoter region of *COL15A1* can be indicative for the down-regulation of transcript levels.

We showed the possibility of detecting novel SNPs (and genes) that do not reach genome-wide statistical significance using conventional GWAS approaches but may confer an increase in risk of disease development. A crucial step in our approach was to relax the traditional GWAS *P*-value threshold (which is *P* < 5 × 10^−8^), which we confidently could do because the *P*-value describes the association with the (SNP) genotype, and not the gene function. Thus, a relatively small phenotypic effect for a SNP can still have large effect on the gene level, particularly, through the presence of deleterious variant(s) in the coding region (as shown in the current work). The effect of such variant(s) might be exacerbated by the presence of aberrant overexpression due to DNA hypomethylation. Conversely, the expression of genes required for normal neurological function is lacking or may be silenced as the transcription is suppressed by DNA hypermethylation. Therefore, we hypothesized that by employing a double-hit model, potential novel targets for brain/neurological functions can be detected. A disadvantage of relaxing the *P*-value threshold is that we may have detected false positive associations with the phenotype. To overcome this, we took various steps to remove genes that are annotated as being spurious^43^, we focused only on the deleterious SNPs that are present in coding regions, and we incorporated the DNA-methylation profiles of the FTD cases. All together we could demonstrate a significant number of genes that harbor both risk SNPs and significant differences in DNA-methylation levels. This indicates non-random behavior of genes that are target in both FTD and FTD-ALS.

For our third aim, we examined whether genetic and epigenetic changes for FTD and/or FTD-ALS may be both present in specific biological processes. One of the pathways that we detected in FTD-ALS with both genetic and epigenetic changes are histone modifications H3k4me3 and H3k27me3, which were previously described to be associated in neurological functions^32^, and involved in social exclusion^44^ by examining liver tissue in mice. Thus overall, evidence is pointing to histone modifications and the association with neurological function. In that perspective, we also demonstrate that this particular pathway is affected in cases with FTD-ALS for both the genetic (SNPs) and epigenetic profiles (DMP). The histone modifications changes are of interest because of their regulation by DNA methyltransferase, such as DNMT3A/B^45,46^, and subsequently for usage of DNMT inhibitor (DNMTi) therapies. The DNMTi targets include azacitidine, and decitabine which are FDA approved for use in leukemia^47^. For neurodegenerative diseases, it may also provide a handle for therapy because cytosine methylation can be targets for DNMTi to reverse the methylation status. A potential candidate gene that we detected can for example be gene *COL15A1*^48^ but this would first require independent replication/validation.

For FTD, single-gene DNA-methylation promoter analysis was performed previously for *MAPT, GRN*, and *C9orf72*. For *MAPT*, no significant differences in DNA-methylation levels were previously seen^36^, whereas both *GRN* and *C9orf72* were shown to contain DNA hypermethylation in the promoter region^11,37^. We expected to see similar results in our analysis but genome-wide DNA-methylation analysis revealed no significance for the probes associated with genes these three genes. A reason for such discrepancy could be that DNA-methylation occurs in specific promoter regions that do not overlap with the Infinium HumanMethylation450 BeadChip probes, which is true for *MAPT* and *GRN* (Table S7).

Our analyses are based on the assumption that the use of DMPs measured in blood is a proxy for DMPs in brain. We carefully examined the proxy, and demonstrate that differential expressed genes in blood, liver, and brain tissue significantly overlapped with the differential expressed genes that are also relevant to FTD-ALS. Although for neurodegenerative diseases, brain would be the preferential tissue to investigate DNA-methylation profiles in, the use of peripheral blood might to some extent overcome this issue as we showed that a significant number of genes with differentially DMPs in the blood are also important for molecular processes in brain. Nonetheless, the use of peripheral blood to analyze DNA-methylation profiles as a model for brain tissue requires caution. Besides the use of blood, other tissues, such as liver, also showed to be representative to examine neurological function as shown in mice^44^, and is in line with our findings.

The DMP data used in this study originates from Li, Y. *et al*.^19^, but we focused specifically on the FTD cases (and not PSP), for which we integratively analyzed the epigenetic and genetic status of genes. In addition, we combined the two batches of samples after batch-correction normalization. This allowed unsupervised analysis using all samples together, and the increased number of samples provided increased statistical power to detect differential methylated genes. Overall, the differential methylated genes from our analysis are in line with those previously detected using the batches separately and in the meta-analysis^19^ (*P*_overlap_ gene set-1: 0.0298, *P*_overlap_ gene set-2: 0.0726, and *P*_overlap_ combined meta-analysis: 0.0073, Fig. S5). Interesting to note is that we detected for the FTD-ALS group in total 224 differential expressed genes, whereas the FTD cases showed only 14 genes, compared to the controls. To accommodate co-variates responsible for changes in methylation that are unrelated to FTD, we analyzed an additional control set of DNA-methylation profiles (GSE53045, Fig. S4A,B) as an alternative approach. We compared the DNA methylated profiles of the controls in the FTD cohort versus the independent control group (non-smokers), which did not yield significance of probes (Fig. S4C). In addition, we compared FTD vs. Controls together with the non-smoker group which resulted in 34 differential DNA methylated probes (Table S8, Fig. S4D). Using this extend control data set, we were able to rule out 2 genes that we initially found to be differential DNA methylated. Note that we already removed these two genes in our final results as the genes were not supported by our incorporated data sources.

The joint analysis and integration of multiple omic data sets is key to further analyze complex neurodegenerative diseases such as FTD. Although our results are based on unpaired samples, by combining genetic and epigenetic data we revealed novel candidate neurodegenerative genes and pathways. Further detailing the biological mechanisms involved in progressive degeneration of the temporal and frontal lobes of the brain requires a well characterized FTD cohort containing clinical, pathological and molecular information for which multi-omic data is obtained for the same samples. With the current work, we showed that both genetic and epigenetic data are useful to start unraveling neurodegenerative processes in FTD.

## Materials and Methods

### GWAS data set

In this study, we used the GWAS summary statistics of 2,154 patients with FTD and separately 200 patients with FTD-ALS^4^. For further analyses, SNPs were retained with unadjusted *P*-value < 0.05 based on the complete FTD cohort and separately for the FTD-ALS cases. SNPs were annotated using ANNOVAR^29^, considered deleterious with CADD-score^49^ >15, and spurious genes were removed^43^.

### DNA-methylation data set processing

The unprocessed beta values (DNA-methylation profiles) were utilized from Li, Y. *et al*. (GEO, accession number GSE53740)^19^. This cohort contains in total 128 FTD cases, of which 118 cases were described with C9orf72 negative status, and 10 cases with a repeat expansion. Seven cases were diagnosed with Amyotrophic Lateral Sclerosis (FTD-ALS) of which 3 cases were *C9orf72* expansion carriers. There were no other reported pathogenic variants in any genes that were screened, including *MAPT* and *GRN*. Prior to making the comparison between FTD cases and controls, we normalized and processed the DNA-methylation beta values to remove technical biases and irrelevant probes (as described below), allowing us to combine the two batches of samples from the original study, instead of performing a meta-analysis by analyzing both batches separately^19^.

The DNA-methylation profiles contained 485,577 probes over 23,179 genes, which were annotated using official Infinium HumanMethylation450 BeadChip annotations. The software package Combat^50^ was used to remove batch effects, allowing us to combine all samples for further analysis instead of performing meta-analysis as previously described^19^. Furthermore, we removed probes that contained > 20% missing values based on all samples. We removed probes that are located on the X and Y chromosome to avoid gender related biases. Furthermore, we removed probes that contain SNPs with MAF > 0.1 (derived from the dbSNP137) as the detection of SNPS that are common in the population can affect DNA-methylation levels and are more likely associated with e.g., ethnicity^51^ instead of disease phenotype. We also removed so-called control probes, and probes that are marked as being spurious^52^. Furthermore, we retained only probes located in close proximity of the annotated gene, i.e., TSS1500, TSS200, 5UTR, 1^st^ Exon, Body, or 3′UTR (based on original Infinium HumanMethylation450 BeadChip annotations). Probes that contained missing values were imputed using the K = 3 nearest neighbor approach. Beta values were zero-mean normalized, i.e., DNA hypermethylation is depicted with relative values above 0 and DNA hypomethylation is depicted with relative values below 0. The final set contained 214,170 probes over 20,956 genes. Currently, various pipelines and packages for Infinium HumanMethylation450 BeadChip processing are developed that can be used for data pre-processing^53^.

### Gene-expression validation data sets

#### Dataset 1

Cell line derived gene expression profiles are utilized that mimic hallmarks of frontotemporal dementias and amyotrophic lateral sclerosis. Processed data is utilized with accession number GSE18632^54^ (Affymetrix Human Genome U133A 2.0 Array), that studied the knockdown of transactive response DNA-binding protein TDP-43 by comparison of 4 controls (HEK293E cells, scrambled) versus 4 KO (HEK293E cells, TDP-43 siRNA). Processed gene expression profiles are log2 transformed. The data set contains in total 54,675 probes over 22,486 unique gene symbols.

#### Dataset 2

For FTD, processed gene expression values (Affymetrix Human Genome U133A 2.0 Array) were utilized from Chen-Plotkin AS. *et al*.^55^ (GEO, accession number GSE13162). This cohort contains in total 56 postmortem human brain samples, among them 39 FTLD-U samples (Frontotemporal lobar degeneration), and 17 control samples. Processed gene expression profiles contain in total 22,277 probes over 13,331 unique gene symbols.

#### Dataset 3

Processed gene expression data is utilized with accession number GSE68605^56^ (Affymetrix Human Genome U133A 2.0 Array), that studied 8 ALS patients with C9orf72 mutations versus 3 neurologically healthy controls. The data set contains in total 54,675 probes over 22,486 unique gene symbols.

#### Dataset 4

Processed gene expression profiles (Affymetrix Human Genome U133 Plus 2.0 Array) were utilized with accession number GSE40438^57^, that studied the selective vulnerability of motor neurons in ALS. This cohort contains samples from 4 oculomotor and 4 lumbar spinal motor neurons which are isolated by laser capture microdissection from the midbrain and spinal cord of neurologically normal human controls. Processed gene expression profiles are log2 transformed, and contains in total 54,675 probes over 22,486 unique gene symbols.

### Tissue-type association

RNA sequencing data, with the expression levels of 16,115 genes, from 1,641 tissue samples over 25 unique tissue types was derived from the GTEx consortium^26,27^. To determine tissue enrichment with the DNA-methylated genes, we followed the procedure as outlined in Fig. 2A. Step 1: for each of the 25 tissue types we tested for differential gene expression between samples within a tissue versus all other tissue samples. Step 2: significantly differentially expressed genes for each tissue type were selected when the absolute Fold-difference > 1.5, and the *P*-value of the Students T-test was ≤ 0.05 after correcting for multiple testing using the Benjamini and Hochberg method. Step 3: the hypergeometric test was applied to determine the significance in overlap between the tissue-type-genes and the DNA-methylated genes in FTD(/ALS) based on the following parameters; total number of genes from GTEx consortium (M = 16,115), number of tissue specific genes (K), number of significant differentially methylated genes (N), and the overlap of significant differentially methylated genes and the genes in the tissue specific gene set (x). The adjusted *P*-value (P*) with < 0.05 was used for tissue selection.

The same procedure was applied for the BrainSpan^28^ data to determine brain-tissue enrichment based on the RNA-sequencing data of 525 samples across 26 brain regions, DNA-methylation data of 177 samples over 17 brain regions, and by using 22 pre-defined gene sets. The pre-defined gene sets describe genes with known function across the various brain regions, and are derived from the official BrainSpan website. As a background, we used the total number of unique genes from Brainspan (RNA-sequencing M = 18,107, and DNA-methylation M = 23,093).

### Pathway/gene set analysis

We utilized the following pathways and gene sets from the molecular signature database (MsigDB v5.1)^31^: chemical and genetic perturbations (n = 3,396), Biocarta genesets (n = 217), KEGG genesets (n = 186), Canonical pathways (n = 1,330), Gene ontology Biological Processes (GO, n = 825), Gene ontology Cellular Components (GO, n = 233), Gene ontology Molecular Function (GO, n = 396), Oncogenic signatures(n = 189), and Immunologic signatures(n = 4,872). To lower the computational burden, we selected a priori for pathways/gene sets with brain or neurological function. Using the hypergeometric test, we calculated a *P*-value for the fraction of genes that overlapped with the annotated pathways/gene sets. A pathway was considered statistically significant when the *P*-value from the hypergeometric test ≤ 0.05 after correcting for multiple testing using the Benjamini and Hochberg method. As a background, we used the M = 25,318 genes from UCSC HG19.

### Co-expression network

The co-expression network is constructed based on pairwise Spearman correlations between the continuous mRNA expression levels using gene expression profiles of the GTEx consortium. For FTD-ALS we started out with the 224 genes and retained 150 genes that overlapped with genes from the GTEx consortium, and that showed a minimum absolute correlation of |r| > 0.6, and significant pairwise interactions *P* < 0.001. Edges with positive correlations are indicated in red (r > 0.6), whereas negative correlations are indicated in blue (r < 0.6). Thickness of edges is based on the absolute correlation measure, |r|, which varies between 0.6 and 1. The gene-degree is determined by the number of edges a gene contains in the co-expression network.

## Electronic supplementary material

Supplementary Materials
