# Author Correction: Susceptible genes and disease mechanisms identified in frontotemporal dementia and frontotemporal dementia with Amyotrophic Lateral Sclerosis by DNA-methylation and GWAS

**DOI:** 10.1038/s41598-018-21308-x

**Published:** 2018-05-14

**Authors:** E. Taskesen, A. Mishra, S. van der Sluis, R. Ferrari, International FTD-Genomics Consortium, D. G. Hernandez, M. A. Nalls, J. D. Rohrer, A. Ramasamy, J. B. J. Kwok, C. Dobson-Stone, P. R. Schofield, G. M. Halliday, J. R. Hodges, O. Piguet, L. Bartley, E. Thompson, E. Haan, I. Hernández, A. Ruiz, M. Boada, B. Borroni, A. Padovani, C. Cruchaga, N. J. Cairns, L. Benussi, G. Binetti, R. Ghidoni, G. Forloni, D. Albani, D. Galimberti, C. Fenoglio, M. Serpente, E. Scarpini, J. Clarimón, A. Lleó, R. Blesa, M. Landqvist Waldö, K. Nilsson, C. Nilsson, I. R. A. Mackenzie, G.-Y. R. Hsiung, D. M. A. Mann, J. Grafman, C. M. Morris, J. Attems, T. D. Griffiths, I. G. McKeith, A. J. Thomas, P. Pietrini, E. D. Huey, E. M. Wassermann, A. Baborie, E. Jaros, M. C. Tierney, P. Pastor, C. Razquin, S. Ortega-Cubero, E. Alonso, R. Perneczky, J. Diehl-Schmid, P. Alexopoulos, A. Kurz, I. Rainero, E. Rubino, L. Pinessi, E. Rogaeva, P. St George-Hyslop, G. Rossi, F. Tagliavini, G. Giaccone, J. B. Rowe, J. C. M. Schlachetzki, J. Uphill, J. Collinge, S. Mead, A. Danek, V. M. Van Deerlin, M. Grossman, J. Q. Trojanowski, J. van der Zee, C. Van Broeckhoven, S. F. Cappa, I. Leber, D. Hannequin, V. Golfier, M. Vercelletto, A. Brice, B. Nacmias, S. Sorbi, S. Bagnoli, I. Piaceri, J. E. Nielsen, L. E. Hjermind, M. Riemenschneider, M. Mayhaus, B. Ibach, G. Gasparoni, S. Pichler, W. Gu, M. N. Rossor, N. C. Fox, J. D. Warren, M. G. Spillantini, H. R. Morris, P. Rizzu, P. Heutink, J. S. Snowden, S. Rollinson, A. Richardson, A. Gerhard, A. C. Bruni, R. Maletta, F. Frangipane, C. Cupidi, L. Bernardi, M. Anfossi, M. Gallo, M. E. Conidi, N. Smirne, R. Rademakers, M. Baker, D. W. Dickson, N. R. Graff-Radford, R. C. Petersen, D. Knopman, K. A. Josephs, B. F. Boeve, J. E. Parisi, W. W. Seeley, B. L. Miller, A. M. Karydas, H. Rosen, J. C. van Swieten, E. G. P. Dopper, H. Seelaar, P. Scheltens, G. Logroscino, R. Capozzo, V. Novelli, A. A Puca, M. Franceschi, A. Postiglione, G. Milan, P. Sorrentino, M. Kristiansen, H.-H. Chiang, C. Graff, F. Pasquier, A. Rollin, V. Deramecourt, T. Lebouvier, D. Kapogiannis, L. Ferrucci, S. Pickering-Brown, A. B. Singleton, J. Hardy, P. Momeni, J. H. Veldink, M. A. van Es, A. B. Smit, D. Posthuma, Y. Pijnenburg

**Affiliations:** 1 VU University Amsterdam, Center for Neurogenomics and Cognitive Research, Complex Trait Genetics (CTG), Amsterdam Neuroscience, Amsterdam, The Netherlands; 2 VU University Medical Center (VUMC), Alzheimer Center, Amsterdam Neuroscience, Amsterdam, The Netherlands; 3 UCL London, Institute of Neurology, Department of Molecular Neuroscience, London, UK; 4 Department of Neurology, Brain Center Rudolf Magnus, University Medical Center Utrecht, Utrecht, The Netherlands; 5 VU University Amsterdam, Center for Neurogenomics and Cognitive Research, Department of Molecular and Cellular Neurobiology (MCN), Amsterdam Neuroscience, Amsterdam, The Netherlands; 6 Laboratory of Neurogenetics, National Institute on Aging, National Institutes of Health, Building 35, Room 1A215, 35 Convent Drive, Bethesda, MD 20892 USA; 7 Dementia Research Centre, Department of Neurodegenerative Disease, UCL Institute of Neurology, London, UK; 8 Department of Medical and Molecular Genetics, King’s College London Tower Wing, Guy’s Hospital, London, SE1 9RT UK; 9 Neuroscience Research Australia, Sydney, NSW 2031 Australia; 10 South Australian Clinical Genetics Service, SA Pathology at Women’s and Children’s Hospital, North Adelaide, SA 5006 Australia; 11 Research Center and Memory Clinic of Fundació ACE, Institut Català de Neurociències Aplicades, Barcelona, Spain; 12 Barbara Borroni (Neurology Clinic, University of Brescia, Brescia, Italy), Alessandro Padovani (Neurology Clinic, University of Brescia, Brescia, Italy; 13 Department of Psychiatry, Washington University, St. Louis, MO USA; 14 Department of Pathology and Immunology, Washington University, St. Louis, MO USA; 15 Molecular Markers Laboratory, IRCCS Istituto Centro San Giovanni di Dio Fatebenefratelli, Brescia, Italy; 16 MAC Memory Clinic, IRCCS Istituto Centro San Giovanni di Dio Fatebenefratelli, Brescia, Italy; 17 Biology of Neurodegenerative Disorders, IRCCS Istituto di Ricerche Farmacologiche, “Mario Negri”, Milano, Italy; 18 University of Milan, Milan, Italy; Fondazione Cà Granda, IRCCS Ospedale Maggiore Policlinico, via F. Sforza 35, 20122 Milan, Italy; 19 Memory Unit, Neurology Department and Sant Pau Biomedical Research Institute, Hospital de la Santa Creu i Sant Pau, Universitat Autònoma de Barcelona, Barcelona, Spain; 20 Unit of Geriatric Psychiatry, Department of Clinical Sciences, Lund University, Lund, Sweden; 21 Clinical Memory Research Unit, Department of Clinical Sciences, Lund University, Lund, Sweden; 22 Department of Pathology and Laboratory Medicine, University of British Columbia, Vancouver, Canada; 23 Division of Neurology, University of British Columbia, Vancouver, Canada; 24 Institute of Brain, Behaviour and Mental Health, University of Manchester, Salford Royal Hospital, Stott Lane, Salford, M6 8HD UK; 25 Department of Psychology, Weinberg College of Arts and Sciences, Northwestern University, Chicago, USA; 26 Newcastle University, Institute of Neuroscience and Institute for Ageing, Campus for Ageing and Vitality, NE4 5PL Newcastle upon Tyne, UK; 27 IMT School for Advanced Studies, Lucca, Lucca, Italy; 28 Taub Institute, Departments of Psychiatry and Neurology, Columbia University, 630 West 168th Street, New York, NY 10032 USA; 29 Behavioral Neurology Unit, National Insititute of Neurological Disorders and Stroke, National Insititutes of Health, 10 CENTER DR MSC 1440, Bethesda, MD 20892-1440 USA; 30 Department of Laboratory Medicine & Pathology, Walter Mackenzie Health Sciences Centre, 8440 - 112 St, University of Alberta Edmonton, Alberta, T6G 2B7 Canada; 31 Department of Neurology, Clínica Universidad de Navarra, University of Navarra School of Medicine, Pamplona, Spain; 32 Neuroepidemiology and Ageing Research Unit, School of Public Health, Faculty of Medicine, The Imperial College of Science, Technology and Medicine, London, W6 8RP UK; 33 Department of Psychiatry and Psychotherapy, Technische Universität München, Munich, 81675 Germany; 34 Neurology I, Department of Neuroscience, University of Torino, Italy, A.O. Città della Salute e della Scienza di Torino, Torino, Italy; 35 Tanz Centre for Research in Neurodegenerative Diseases, University of Toronto 60 Leonard Street, Toronto, Ontario, Canada M5T 2S8 USA; 36 Division of Neurology V and Neuropathology, Fondazione IRCCS Istituto Neurologico Carlo Besta, 20133 Milano, Italy; 37 Cambridge University Department of Clinical Neurosciences, Cambridge, CB2 0SZ UK; 38 University of California San Diego, Department of Cellular & Molecular Medicine, 9500 Gilman Drive, La Jolla, CA 92093 USA; 39 MRC Prion Unit, Department of Neurodegenerative Disease, UCL Institute of Neurology, Queen Square House, WC1N 3BG Queen Square, London UK; 40 Neurologische Klinik und Poliklinik, Ludwig-Maximilians-Universität, German Center for Neurodegenerative Diseases (DZNE), Munich, Germany; 41 University of Pennsylvania Perelman School of Medicine, Department of Pathology and Laboratory Medicine, Philadelphia, PA USA; 42 Neurodegenerative Brain Diseases group, VIB-UAntwerp Center of Molecular Neurology, Antwerp, Belgium; Laboratory of Neurogenetics, Institute Born-Bunge, University of Antwerp, Antwerp, Belgium; 43 Neurorehabilitation Unit, Dept. Of Clinical Neuroscience, Vita-Salute University and San Raffaele Scientific Institute, Milan, Italy; 44 Inserm, UMR_S975, CRICM; UPMC Univ Paris 06, UMR_S975; CNRS UMR 7225, F-75013, Paris, France; AP-HP, Hôpital de la Salpêtrière, Département de neurologie-centre de références des démences rares, F-75013 Paris, France; 45 Service de Neurologie, Inserm U1079, CNR-MAJ, Rouen University Hospital, Rouen, France; 46 Department of Neurosciences, Psychology, Drug Research and Child Health (NEUROFARBA) University of Florence, Florence, Italy; 47 Danish Dementia Research Centre, Neurogenetics Clinic, Department of Neurology, Rigshospitalet, Copenhagen University Hospital, Copenhagen, Denmark; 48 Saarland University, Laboratory for Neurogenetics, Kirrberger Str.1, Bld.90, 66421 Homburg/Saar, Germany; 49 University Regensburg, Department of Psychiatry, Psychotherapy and Psychosomatics, Universitätsstr. 84, 93053 Regensburg, Germany; 50 University of Cambridge, Department of Clinical Neurosciences, John Van Geest Brain Repair Centre, Forvie Site, Robinson way, Cambridge, CB2 0PY UK; 51 German Center for Neurodegenerative Diseases-Tübingen, Otfried Muellerstrasse 23, Tuebingen, 72076 Germany; 52 Regional Neurogenetic Centre, ASPCZ, Lamezia Terme, Italy; 53 Department of Neuroscience, Mayo Clinic Jacksonville, 4500 San Pablo Road, Jacksonville, FL 32224 USA; 54 Department of Neurology, Mayo Clinic Rochester, 2001st street SW, Rochester, MN 5905 USA; 55 William W Seeley (Department of Neurology, Box 1207, University of California, San Francisco, CA 94143 USA; 56 Memory and Aging Center, Department of Neurology, University of California, San Francisco, CA 94158 USA; 57 Department of Neurology, Erasmus Medical Centre, Rotterdam, The Netherlands; 58 Alzheimer Centre and department of neurology, VU University medical centre, Amsterdam, The Netherlands; 59 Department of Basic Medical Sciences, Neurosciences and Sense Organs of the “Aldo Moro” University of Bari, Bari, Italy; 60 Medical Genetics Unit, Fondazione Policlinico Universitario A. Gemelli, Rome, Italy; 61 Cardiovascular Research Unit, IRCCS Multimedica, Milan, Italy; 62 Neurology Dept, IRCCS Multimedica, Milan, Italy; 63 Department of Clinical Medicine and Surgery, University of Naples Federico II, Naples, Italy; 64 UCL Genomics, Institute of Child Health (ICH), UCL, London, UK; 65 Karolinska Institutet, Dept NVS, Alzheimer Research Center, Novum, SE-141 57 Stockholm, Sweden; 66 Univ Lille, Inserm 1171, DISTALZ, CHU 59000 Lille, France; 67 National Institute on Aging (NIA/NIH), 3001 S. Hanover St, NM 531, Baltimore, MD 21230 USA; 68 Clinical Research Branch, National Institute on Aging, Baltimore, MD USA; 69 Laboratory of Neurogenetics, Department of Internal Medicine, Texas Tech University Health Science Center, 4th street, Lubbock, Texas 79430 USA

Correction to: *Scientific Reports* 10.1038/s41598-017-09320-z, published online 21 August 2017

International FTD-Genomics Consortium was omitted from the author list in the original version of this Article. This has been corrected in the PDF and HTML versions of the Article, and in the accompanying Supplementary Material file.

The Acknowledgements section now reads:

“We thank the International FTD-Genomics Consortium (IFGC) for providing the data of the FTD and FTD-MND summary statistics. The acknowledgments for and the consortia members of the IFGC are shown below. We furthermore thank G. Coppola and D.H. Geschwind for providing information of the DNA-methylation FTD patient cohort that is used in this study. We thank Ingrosyl for their financial support to perform this study. SvdS is funded by the Netherlands Scientific Organization (NWO/MaGW: VIDI-452-12-014). Raffaele Ferrari is supported by Alzheimer’s Society Grant 284.

Intramural funding from the National Institute of Neurological Disorders and Stroke (NINDS) and National Institute on Aging (NIA), the Wellcome/MRC Centre on Parkinson’s disease, Alzheimer’s Research UK (ARUK, Grant ARUK-PG2012-18) and by the office of the Dean of the School of Medicine, Department of Internal Medicine, at Texas Tech University Health Sciences Center. We thank Mike Hubank and Kerra Pearce at the Genomic core facility at the Institute of Child Health (ICH), University College of London (UCL), for assisting RF in performing Illumina genotyping experiments (FTD-GWAS genotyping). This study utilized the high-performance computational capabilities of the Biowulf Linux cluster at the National Institutes of Health, Bethesda, Md. (http://biowulf.nih.gov). North American Brain Expression Consortium (NABEC) - The work performed by the North American Brain Expression Consortium (NABEC) was supported in part by the Intramural Research Program of the National Institute on Aging, National Institutes of Health, part of the US Department of Health and Human Services; project number ZIA AG000932-04. In addition this work was supported by a Research Grant from the Department of Defense, W81XWH-09-2-0128. UK Brain Expression Consortium (UKBEC) - This work performed by the UK Brain Expression Consortium (UKBEC) was supported by the MRC through the MRC Sudden Death Brain Bank (C.S.), by a Project Grant (G0901254 to J.H. and M.W.) and by a Fellowship award (G0802462 to M.R.). D.T. was supported by the King Faisal Specialist Hospital and Research Centre, Saudi Arabia. Computing facilities used at King’s College London were supported by the National Institute for Health Research (NIHR) Biomedical Research Centre based at Guy’s and St Thomas’ NHS Foundation Trust and King’s College London. We would like to thank AROS Applied Biotechnology AS company laboratories and Affymetrix for their valuable input. RF’s work is supported by Alzheimer’s Society (grant number 284), UK; JBJK was supported by the National Health and Medical Resarch Council (NHMRC) Australia, Project Grants 510217 and 1005769; CDS was supported by NHMRC Project Grants 630428 and 1005769; PRS was supported by NHMRC Project Grants 510217 and 1005769 and acknowledges that DNA samples were prepared by Genetic Repositories Australia, supported by NHMRC Enabling Grant 401184; GMH was supported by NHMRC Research Fellowship 630434, Project Grant 1029538, Program Grant 1037746; JRH was supported by the Australian Research Council Federation Fellowship, NHMRC Project Grant 1029538, NHMRC Program Grant 1037746; OP was supported by NHMRC Career Development Fellowship 1022684, Project Grant 1003139. IH, AR and MB acknowledge the patients and controls who participated in this project and the Trinitat Port-Carbó and her family who are supporting Fundació ACE research programs. CC was supported by Grant P30-NS069329-01 and acknowledges that the recruitment and clinical characterization of research participants at Washington University were supported by NIH P50 AG05681, P01 AG03991, and P01 AG026276. LB and GB were supported by the Ricerca Corrente, Italian Ministry of Health; RG was supported by Fondazione CARIPLO 2009-2633, Ricerca Corrente, Italian Ministry of Health; GF was supported by Fondazione CARIPLO 2009-2633. ES was supported by the Italian Ministry of Health; CF was supported by Fondazione Cariplo; MS was supported from the Italian Ministry of Health (Ricerca Corrente); MLW was supported by Government funding of clinical research within NHS Sweden (ALF); KN was supported by Thure Carlsson Foundation; CN was supported by Swedish Alzheimer Fund. IRAM and GYRH were supported by CIHR (grant 74580) PARF (grant C06-01). JG was supported by the NINDS intramural research funds for FTD research. CMM was supported by Medical Research Council UK, Brains for Dementia Research, Alzheimer’s Society, Alzheimer’s Research UK, National Institutes for Health Research, Department of Health, Yvonne Mairy Bequest and acknowledges that tissue made available for this study was provided by the Newcastle Brain Tissue Resource, which was funded in part by grants G0400074 and G1100540 from the UK MRC, the Alzheimer’s Research Trust and Alzheimer’s Society through the Brains for Dementia Research Initiative and an NIHR Biomedical Research Centre Grant in Ageing and Health, and NIHR Biomedical Research Unit in Lewy Body Disorders. CMM was supported by the UK Department of Health and Medical Research Council and the Research was supported by the National Institute for Health Research Newcastle Biomedical Research Centre based at Newcastle Hospitals Foundation Trust and Newcastle University and acknowledges that the views expressed are those of the authors and not necessarily those of the NHS, the NIHR or the Department of Health; JA was supported by MRC, Dunhill Medical Trust, Alzheimer’s Research UK; TDG was supported by Wellcome Trust Senior Clinical Fellow; IGM was supported by NIHR Biomedical Research Centre and Unit on Ageing Grants and acknowledges the National Institute for Health Research Newcastle Biomedical Research Centre based at Newcastle Hospitals Foundation Trust and Newcastle University. The views expressed are those of the author(s) and not necessarily those of the NHS, the NIHR or the Department of Health; AJT was supported by Medical Research Council, Alzheimer’s Society, Alzheimer’s Research UK, National Institutes for Health Research. EJ was supported by NIHR, Newcastle Biomedical Research Centre. PP, CR, SOC and EA were supported partially by FIMA (Foundation for Applied Medical Research); PP acknowledges Manuel Seijo-Martínez (Department of Neurology, Hospital do Salnés, Pontevedra, Spain), Ramon Rene, Jordi Gascon and Jaume Campdelacreu (Department of Neurology, Hospital de Bellvitge, Barcelona, Spain) for providing FTD DNA samples. RP, JDS, PA and AK were supported by German Federal Ministry of Education and Research (BMBF; grant number FKZ 01GI1007A – German FTLD consortium). IR was supported by Ministero dell’Istruzione, dell’Università e della Ricerca (MIUR) of Italy. PStGH was supported by the Canadian Institutes of Health Research, Wellcome Trust, Ontario Research Fund. FT was supported by the Italian Ministry of Health (ricerca corrente) and MIUR grant RBAP11FRE9; GR and GG were supported by the Italian Ministry of Health (ricerca corrente). JBR was supported by Camrbidge NIHR Biomedical Research Centre and Wellcome Trust (088324). JU, JC, SM were supported by the MRC Prion Unit core funding and acknowledge MRC UK, UCLH Biomedical Research Centre, Queen Square Dementia BRU; SM acknowledges the work of John Beck, Tracy Campbell, Gary Adamson, Ron Druyeh, Jessica Lowe, Mark Poulter. AD acknowledges the work of Benedikt Bader and of Manuela Neumann, Sigrun Roeber, Thomas Arzberger and Hans Kretzschmar†; VMVD and JQT were supported by Grants AG032953, AG017586 and AG010124; MG was supported by Grants AG032953, AG017586, AG010124 and NS044266; VMVD acknowledges EunRan Suh, PhD for assistance with sample handling and Elisabeth McCarty-Wood for help in selection of cases; JQT acknowledges Terry Schuck, John Robinson and Kevin Raible for assistance with neuropathological evaluation of cases. CVB and the Antwerp site were in part funded by the MetLife Foundation for Medical Research Award (to CVB), the Belgian Science Policy Office (BELSPO) Interuniversity Attraction Poles program; the Alzheimer Research Foundation (SAO-FRA); the Medical Foundation Queen Elisabeth (GSKE); the Flemish Government initiated Methusalem Excellence Program (to CVB); the Research Foundation Flanders (FWO) and the University of Antwerp Research Fund.. CVB, MC and JvdZ acknowledge the neurologists S Engelborghs, PP De Deyn, A Sieben, R Vandenberghe and the neuropathologist JJ Martin for the clinical and pathological diagnoses. CVB, MC and JvdZ further thank the personnel of the Genetic Service Facility of the VIB Department of Molecular Genetics (http://www.vibgeneticservicefacility.be) and the Antwerp Biobank of the Institute Born-Bunge for their expert support. IL and AB were supported by the program “Investissements d’avenir” ANR-10-IAIHU-06 and acknowledges the contribution of The French research network on FTLD/FTLD-ALS for the contribution in samples collection. BN is founded by Fondazione Cassa di Risparmio di Pistoia e Pescia (grant 2014.0365), SS is founded by the Cassa di Risparmio di Firenze (grant 2014.0310) and a grant from Ministry of Health n° RF-2010-2319722. JEN was supported by the Novo Nordisk Foundation, Denmark. MR was supported by the German National Genome Network (NGFN); German Ministry for Education and Research Grant Number 01GS0465. JDR, MNR, NCF and JDW were supported by an MRC programme grant and the Dementia Platform UK, the NIHR Queen Square Dementia Biomedical Research Unit (BRU) and the Leonard Wolfson Experimental Neurology Centre. MGS was supported by MRC grant n G0301152, Cambridge Biomedical Research Centre and acknowledges Mrs K Westmore for extracting DNA. HM was supported by the Motor Neuron Disease Association (Grant 6057). RR was supported by P50 AG016574, R01 NS080882, R01 NS065782, P50 NS72187 and the Consortium for Frontotemporal Dementia; DWD was supported by P50NS072187, P50AG016574, State of Florida Alzheimer Disease Initiative, & CurePSP, Inc.; NRGR, JEP, RCP, DK, BFB were supported by P50 AG016574; KAJ was supported by R01 AG037491; WWS was supported by NIH AG023501, AG019724, Consortium for Frontotemporal Dementia Research; BLM was supported by P50AG023501, P01AG019724, Consortium for FTD Research; HR was supported by AG032306. JCvS was supported by Stichting Dioraphte Foundation (11 02 03 00), Nuts Ohra Foundation (0801-69), Hersenstichting Nederland (BG 2010-02) and Alzheimer Nederland. CG and HHC acknowledge families, patients, clinicians including Dr Inger Nennesmo and Dr Vesna Jelic, Professor Laura Fratiglioni for control samples and Jenny Björkström, Håkan Thonberg, Charlotte Forsell, Anna-Karin Lindström and Lena Lilius for sample handling. CG was supported by Swedish Brain Power (SBP), the Strategic Research Programme in Neuroscience at Karolinska Institutet (StratNeuro), the regional agreement on medical training and clinical research (ALF) between Stockholm County Council and Karolinska Institutet, Swedish Alzheimer Foundation, Swedish Research Council, Karolinska Institutet PhD-student funding, King Gustaf V and Queen Victoria’s Free Mason Foundation. FP, AR, VD and FL acknowledge Labex DISTALZ. RF acknowledges the help and support of Mrs. June Howard at the Texas Tech University Health Sciences Center Office of Sponsored Programs for tremendous help in managing Material Transfer Agreement at TTUHSC.”

The Author Contributions section now reads:

“E.T. and Y.P. designed the study. E.T. analyzed the data, and drafted the manuscript. E.T., A.M., R.F., J.H.V., M.A.E., A.B.S., D.P., and Y.P. participated in the discussions, interpretation and editing of the manuscript. All authors provided relevant input at different stages of the project and approved the final manuscript. The International FTD-Genomics Consortium (IFGC) provided summary statistics data used to perform the analyses presented in the current work.”

