# Supplementary material for: Susceptible genes and disease mechanisms identified in frontotemporal dementia and frontotemporal dementia with Amyotrophic Lateral Sclerosis by DNA-methylation and GWAS
Source: Sci Rep. 2017 Aug 21;7:8899. doi: 10.1038/s41598-017-09320-z (PMC5567187; doi:10.1038/s41598-017-09320-z)
Supplement: Supplementary file 1 — Supplementary Materials [file 41598_2017_9320_MOESM1_ESM.pdf]

Supplementary materials

Susceptible genes and disease mechanisms identified in frontotemporal dementia and frontotemporal dementia with Amyotrophic Lateral Sclerosis by DNA-methylation and GWAS.

E. Taskesen[1,2], A. Mishra[1], S. van der Sluis[1], R. Ferrari[3], International FTD-Genomics consortium (IFGC), J.H. Veldink[4], M.A. van Es[4], A.B. Smit[5], D. Posthuma[1,2], Y. Pijnenburg[2]*

Affiliations:
[1] VU University Amsterdam, Center for Neurogenomics and Cognitive Research, Complex Trait Genetics (CTG), Amsterdam Neuroscience, Amsterdam, the Netherlands
[2] VU University Medical Center (VUMC), Alzheimer Center, Amsterdam Neuroscience, Amsterdam, the Netherlands
[3] UCL London, Institute of Neurology, Department of Molecular Neuroscience, UK
[4] Department of Neurology, Brain Center Rudolf Magnus, University Medical Center Utrecht, Utrecht, The Netherlands
[5] VU University Amsterdam, Center for Neurogenomics and Cognitive Research, Department of Molecular and Cellular Neurobiology (MCN), Amsterdam Neuroscience, Amsterdam, the Netherlands

* Corresponding author
The authors disclose no potential conflicts of interest

Dr. Erdogan Taskesen	e.taskesen@vu.nl
Dr. Aniket Mishra	a.mishra@vu.nl
Dr. Sophie van der Sluis	s.sluis@vu.nl
Dr. Raffaele Ferrari	r.ferrari@ucl.ac.uk
IFGC	IFGCadm@gmail.com
Prof. Jan H. Veldink	J.H.Veldink@umcutrecht.nl
MD. PhD. Michael A. van Es	M.A.vanEs@umcutrecht.nl
Prof. Guus A.B. Smit	guus.smit@vu.nl
Prof. Danielle Posthuma	danielle.posthuma@vu.nl
MD. PhD. Yolande Pijnenburg	YAL.Pijnenburg@vumc.nl

SUPPLEMENTARY FILES

Fig.S1. Differential DNA-methylated genes and PCA for FTD. (A) P-value distribution plot for the comparison FTD versus Control using Student T-test. (B) Volcano plot depicting genes with the fold-difference on the x-axis and -log10(*P*-value) on the y-axis. (C) Principal Component analysis on the top 20% probes with highest variance.

Fig.S2. Differential DNA-methylated genes and PCA for FTD-ALS. (A) P-value distribution plot for the comparison FTD-ALS versus Control using Student T-test. (B) Volcano plot depicting genes with the fold-difference on the x-axis and -log10(*P*-value) on the y-axis.

Fig.S3. GTEx tissue enrichment for the differential DNA-methylated genes in FTD-ALS. Tissue enrichment was computed by following the approach as outlined in Fig.2A. (A) Tissue enrichment for FTD-ALS with the 25 GTEx specific tissues. (B) Brain tissue enrichment for FTD-ALS with the 13 GTEx specific brain tissues. Genes are colored with the tissue specific color.

Fig.S4. Batch correction and differential DNA methylated genes after extending control data set. (A) batch effect of samples without correction. (B) Clustering of samples after batch-effect correction. (C) Differential DNA methylated probes for Controls (this study) vs. Non-smokers. (D) Differential DNA methylated probes for FTD vs. Non-smokers.

Fig.S5. Venn diagram depicting overlap of genes from the original meta-analysis. Genes from the meta-analysis from the original study were gathered and overlaid with the differential methylated that we detected in this study.

Figure S1

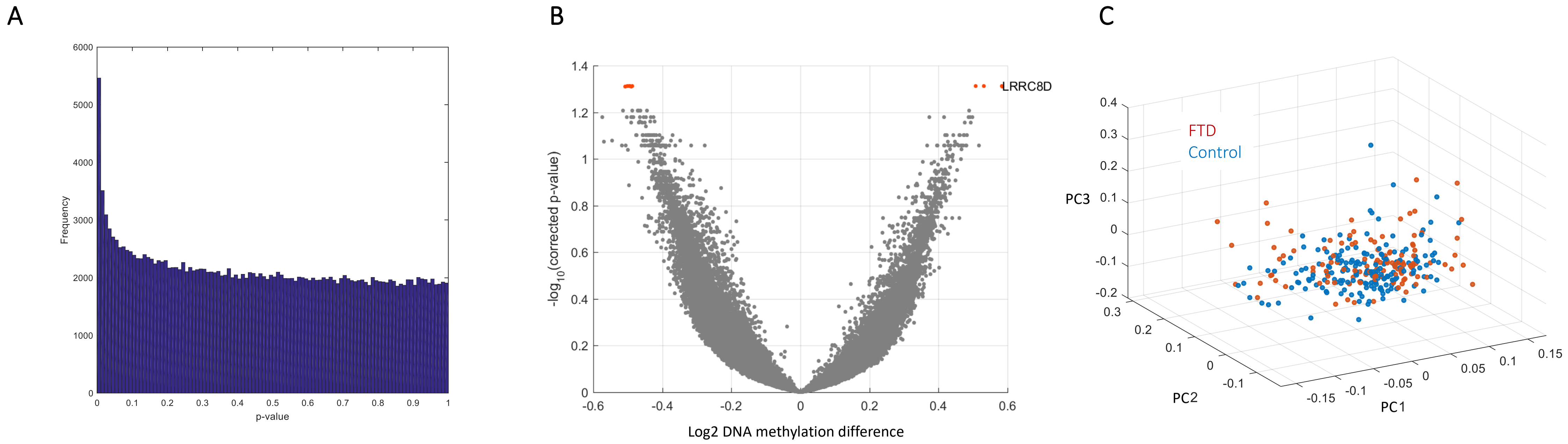
A
Frequency
p-value
B
-log10(corrected p-value)
Log2 DNA methylation difference
LRRC8D
C
FTD
Control
PC1
PC2
PC3

Figure S2

A

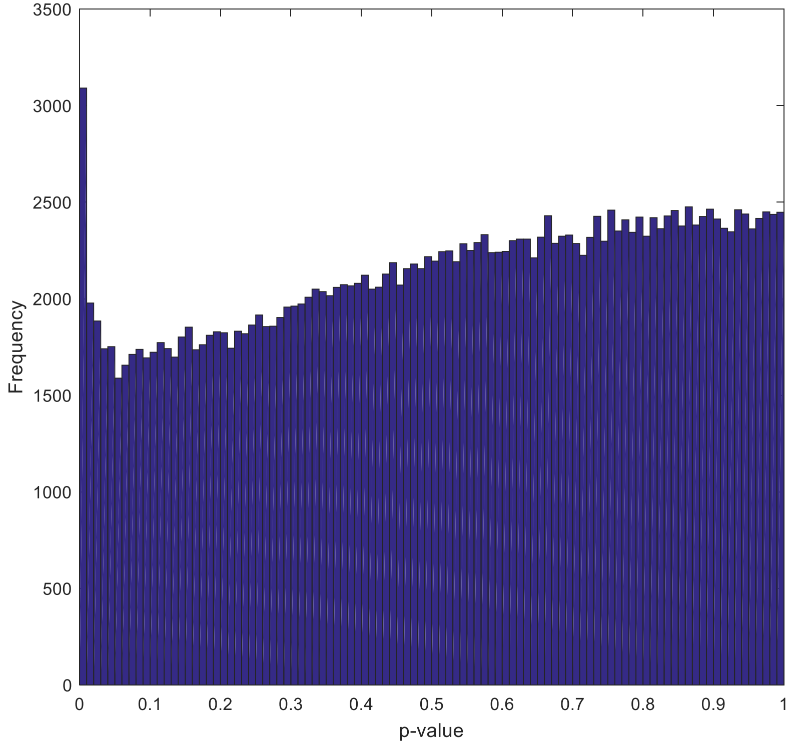
Frequency
p-value
3500
3000
2500
2000
1500
1000
500
0
0.1
0.2
0.3
0.4
0.5
0.6
0.7
0.8
0.9
1

B

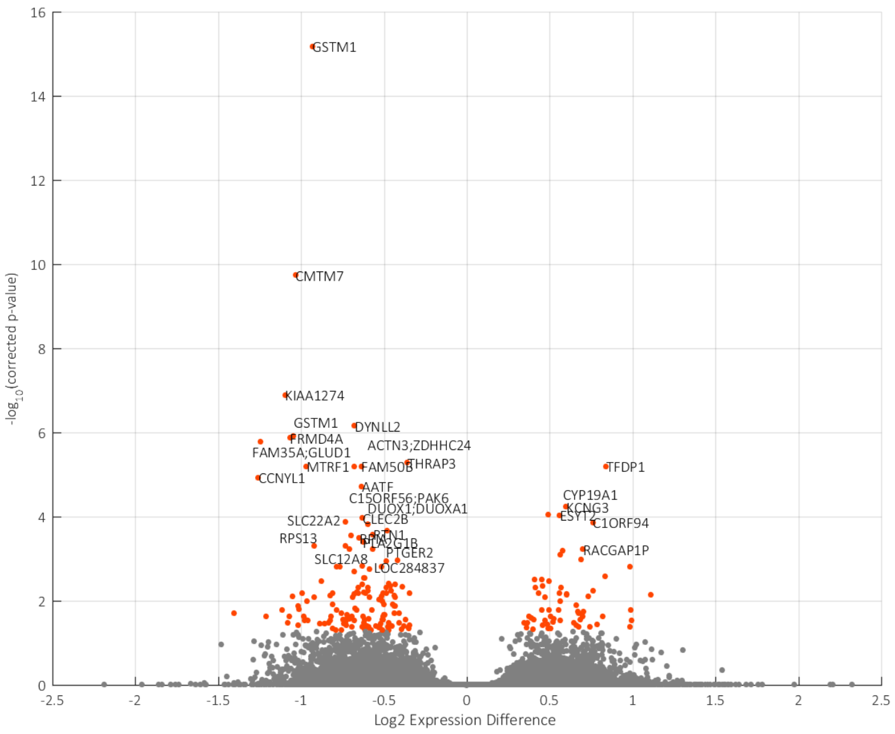
-log10(corrected p-value)
Log2 Expression Difference
GSTM1
CMTM7
KIAA1274
GSTM1
DYNLL2
FRMD4A
ACTN3;ZDHHC24
FAM35A;GLUD1
MTRF1
FAM50B
THRAP3
TFDP1
CCNYL1
AATF
C15ORF56;PAK6
CYP19A1
DUOX1;DUOXA1
KCNG3
SLC22A2
CLEC2B
ESYT2
C1ORF94
RPS13
PTGER2
RACGAP1P
SLC12A8
LOC284837

Figure S3

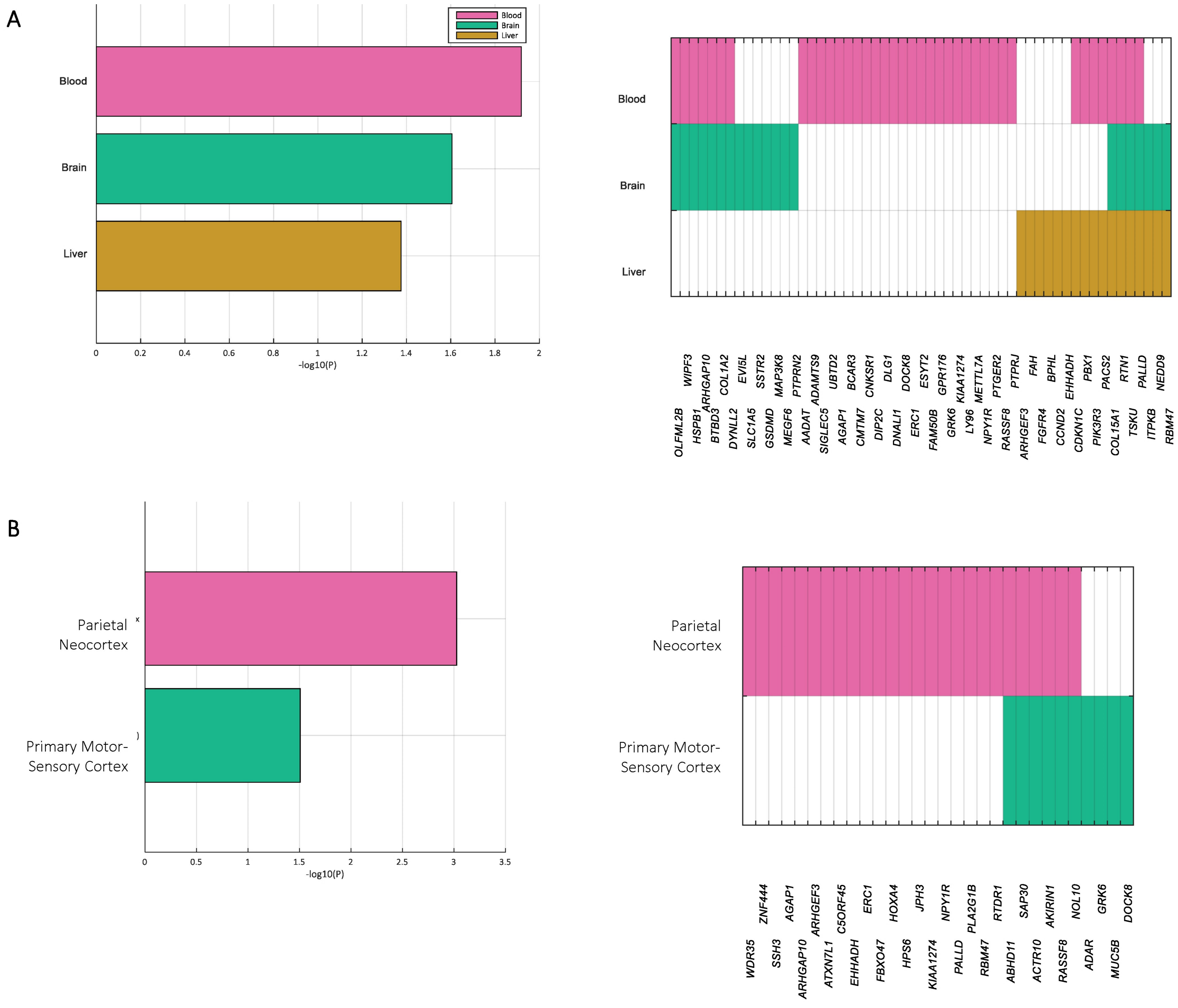
A
Blood
Brain
Liver
0
0.2
0.4
0.6
0.8
1
1.2
1.4
1.6
1.8
2
-log10(P)
OLFML2B
WIPF3
HSPB1
ARHGAP10
BTBD3
COL1A2
DYNLL2
EVI5L
SLC1A5
SSTR2
GSDMD
MAP3K8
MEGF6
PTPRN2
AADAT
ADAMTS9
SIGLEC5
UBTD2
AGAP1
BCAR3
CMTM7
CNKSR1
DIP2C
DLG1
DNALI1
DOCK8
ERC1
ESYT2
FAM50B
GPR176
GRK6
KIAA1274
LY96
METTL7A
NPY1R
PTGER2
RASSF8
PTPRJ
ARHGEF3
FAH
FGFR4
BPHL
CCND2
EHHADH
CDKN1C
PBX1
PIK3R3
PACS2
COL15A1
RTN1
TSKU
PALLD
ITPKB
NEDD9
RBM47
B
Parietal
Neocortex
Primary Motor-
Sensory Cortex
0
0.5
1
1.5
2
2.5
3
3.5
-log10(P)
WDR35
ZNF444
SSH3
AGAP1
ARHGAP10
ARHGEF3
ATXN7L1
C5ORF45
EHHADH
ERC1
FBXO47
HOXA4
HPS6
JPH3
KIAA1274
NPY1R
PALLD
PLA2G1B
RBM47
RTDR1
ABHD11
SAP30
ACTR10
AKIRIN1
RASSF8
NOL10
ADAR
GRK6
MUC5B
DOCK8

Figure S4

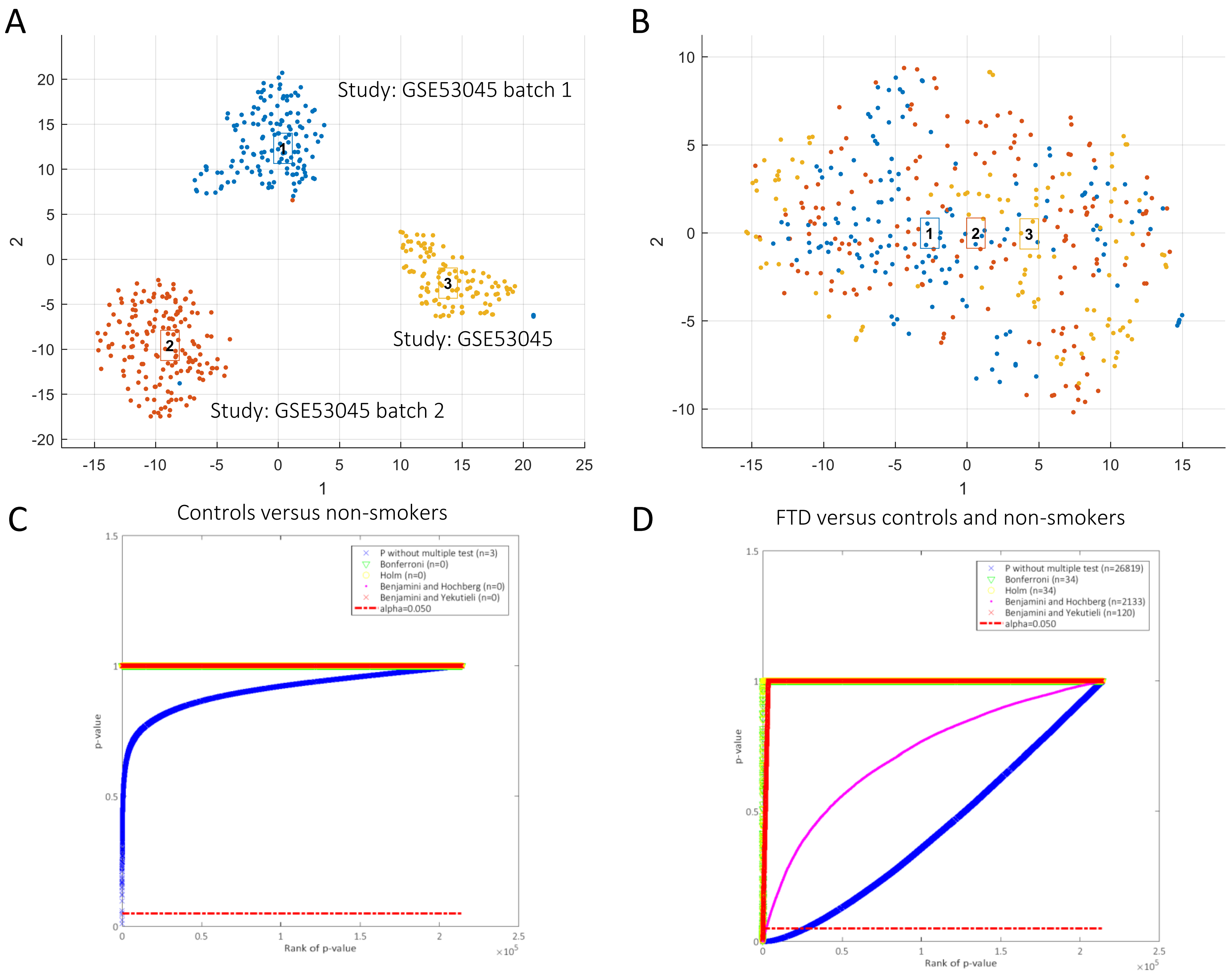

A
Study: GSE53045 batch 1
Study: GSE53045
Study: GSE53045 batch 2
B
C
Controls versus non-smokers
P without multiple test (n=3)
Bonferroni (n=0)
Holm (n=0)
Benjamini and Hochberg (n=0)
Benjamini and Yekutieli (n=0)
alpha=0.050
p-value
Rank of p-value
D
FTD versus controls and non-smokers
P without multiple test (n=26819)
Bonferroni (n=34)
Holm (n=34)
Benjamini and Hochberg (n=2133)
Benjamini and Yekutieli (n=120)
alpha=0.050
p-value
Rank of p-value

Figure S5

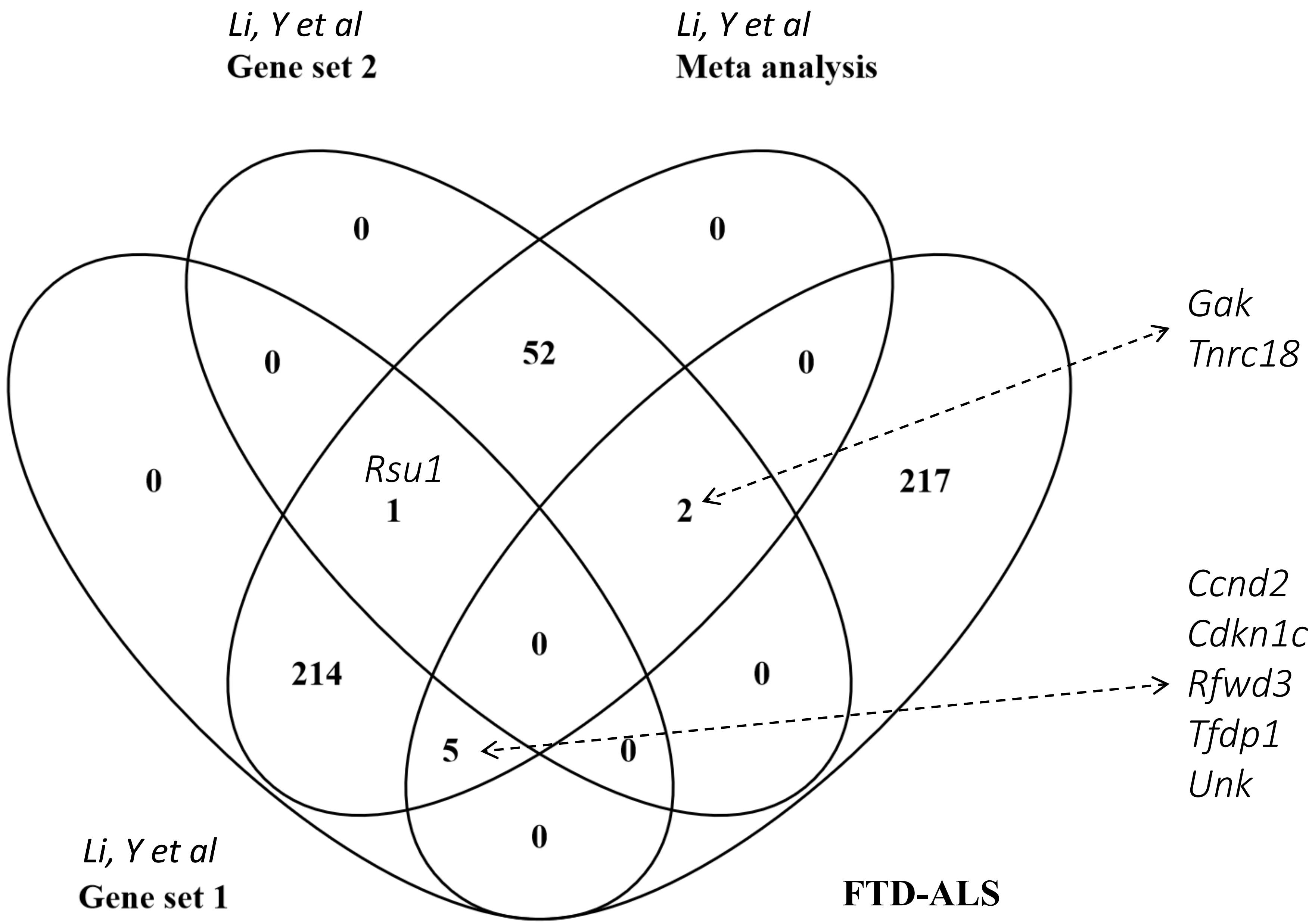
Li, Y et al
Gene set 2
Li, Y et al
Meta analysis
0
0
0
52
0
0
Rsu1
1
2
217
Gak
Tnrc18
0
214
5
0
0
0
Ccnd2
Cdkn1c
Rfwd3
Tfdp1
Unk
Li, Y et al
Gene set 1
FTD-ALS

Table S1

Genesymbol	Chr	Strand	GWAS SNPs						DNA-methylation probes					Description		Validated by gene-expression
			Id	*P*	Location	Mutation type	CADD score	Min(P)	Id	P_{BH}	Location	T-stat	Hyper/hypo	Phenotype	Wikigene	
ELP2/ SLC39A6	chr18	.	rs16967474	0.03221	exonic	nonsynonymous SNV	25.3	0.03221	cg12339809	0.0486004	Island	-5.0795263	Hypomethylation	.	.	Yes
ZMIZ1	chr10	.	rs12571751	0.03573	intronic	.	.	0.03573	cg19168578	0.0486004	Island	-4.9091067	Hypomethylation	.	.	
AP3M1	chr10	-	exm954373	0.01207	intronic	.	.	0.01207	cg04760117	0.0486004	.	4.9137135	Hypermethylation	.	adaptor-related protein complex 3, mu 1 subunit	
ZNF808,ZNF701	chr19	+	exm2216491	0.02767	intergenic	.	.	0.02767	cg10782349	0.0489753	Island	4.9227971	Hypermethylation	.	zinc finger protein 701	
TPK1	chr7	-	.	.	.	.	.	.	cg16316507	0.0486004	Island	-4.9587098	Hypomethylation	THIAMINE METABOLISM DYSFUNCTION SYNDROME 5	thiamin pyrophosphokinase 1	
STK39	chr2	-	.	.	.	.	.	.	cg03159329	0.0486004	Island	-4.9394391	Hypomethylation	.	serine threonine kinase 39	
BMS1P4	chr10	-	.	.	.	.	.	.	cg26771998	0.0489753	Island	-4.8163424	Hypomethylation	.	BMS1 pseudogene 4	
NCRNA00188;SNORD49A;SNORD4	chr17	.	.	.	.	.	.	.	cg02830903	0.0489753	Island	-4.8184838	Hypomethylation	.	.	
LRRC8D	chr1	+	.	.	.	.	.	.	cg01533966	0.0486004	.	4.9315597	Hypermethylation	.	leucine rich repeat containing 8 family, member D	
RAI1	chr17	+	.	.	.	.	.	.	cg19285752	0.0486004	N_Shore	4.8937517	Hypermethylation	Smith-Magenis syndrome (SMS)	retinoic acid induced 1	

Table S2

Genesymbol	Chr	Strand	GWAS SNPs						DNA-methylation probes				Validated by gene-expression
			Id	*P*	Location	Mutation type	CADD score	min(*P*) for gene	Id	P_{BY}	Location	Tstat	
PCDHA4	chr5		rs59233330	1.92E-04	exonic	nonsynonymous SNV	0	1.92E-04	cg02357321	3.348E-02	N_Shore	-6.43	
DLG1	chr3	-	rs74674649	6.01E-04	exonic	nonsynonymous SNV	28	6.01E-04	cg12594803	2.879E-02	.	-7.04	Yes
KIAA1147	chr7	-	rs201876806	9.13E-04	exonic	nonsynonymous SNV	23	9.13E-04	cg24662653	2.890E-03	Island	-6.51	Yes
HPS6	chr10	+	rs36078476	1.07E-03	exonic	nonsynonymous SNV	0	1.07E-03	cg02840794	3.383E-03	Island	-7.36	
GPN3	chr12		rs143166802	1.69E-03	exonic	nonsynonymous SNV	2	1.69E-03	cg00558990	1.524E-02	N_Shore	-5.99	
GGA1	chr22	+	rs143909159	2.91E-03	exonic	nonsynonymous SNV	34	2.91E-03	cg21268578	2.796E-02	.	-7.16	
PTPRN2	chr7	-	rs142437536	3.02E-03	exonic	nonsynonymous SNV	0	3.02E-03	cg05135176	7.528E-03	S_Shore	-5.52	Yes
PPP1R36,PLEKHG3	chr14	+	rs117300689	3.29E-03	intergenic	.	.	3.29E-03	cg26763365	4.418E-02	Island	-5.55	
MRPL21	chr11		rs200690672	3.96E-03	exonic	nonsynonymous SNV	9	3.96E-03	cg26065952	2.314E-02	N_Shore	-5.70	Yes
IGHMBP2	chr11		rs201692151	4.00E-03	exonic	nonsynonymous SNV	23	4.00E-03	cg26065952	2.314E-02	N_Shore	-5.70	Yes
MTRF1	chr13	-	rs9532758	5.25E-03	exonic	nonsynonymous SNV	7	5.25E-03	cg21446692	6.388E+08	.	-10.38	
ASPM	chr1	-	rs150125249	5.78E-03	exonic	nonsynonymous SNV	34	5.78E-03	cg11336294	2.729E-02	.	-5.59	
NEDD9	chr6	-	rs112742585	5.85E-03	exonic	nonsynonymous SNV	13	5.85E-03	cg05917225	9.185E-03	.	-5.79	
PRSS36	chr16	-	rs145749002	6.04E-03	exonic	nonsynonymous SNV	33	6.04E-03	cg14301190	4.949E-03	Island	-6.68	
PPP1R36,PLEKHG3	chr14	+	rs117434243	6.48E-03	intergenic	.	.	3.29E-03	cg26763365	4.418E-02	Island	-5.55	
IGHMBP2	chr11		rs622082	7.05E-03	exonic	nonsynonymous SNV	0	4.00E-03	cg26065952	2.314E-02	N_Shore	-5.70	Yes
PKNOX1	chr21	+	rs2839627	8.18E-03	intronic	.	.	8.18E-03	cg04193427	4.013E-02	Island	-5.38	Yes
CNKSR1	chr1	+	rs144396219	8.32E-03	exonic	nonsynonymous SNV	28	8.32E-03	cg09890400	1.962E-02	.	-8.83	Yes
UBTD2	chr5	-	rs17074452	9.54E-03	exonic	nonsynonymous SNV	18	9.54E-03	ch.5.3268483F	4.949E-03	.	-5.84	Yes
GGA1	chr22	+	rs138525343	9.76E-03	exonic	nonsynonymous SNV	27	2.91E-03	cg21268578	2.796E-02	.	-7.16	
IGHMBP2	chr11		rs145226920	0.01001	exonic	stopgain	35	4.00E-03	cg26065952	2.314E-02	N_Shore	-5.70	Yes
FAH	chr15	+	rs144234072	1.07E-02	exonic	nonsynonymous SNV	27	0.01066	cg06856840	1.648E-02	.	-6.95	
RBM14	chr11	+	rs148119991	1.28E-02	exonic	nonsynonymous SNV	5	0.01281	cg03249986	1.005E-02	Island	-5.46	
NEDD9	chr6	-	rs34044517	1.63E-02	exonic	nonsynonymous SNV	24	5.85E-03	cg05917225	9.185E-03	.	-5.79	
FAH	chr15	+	rs34749737	0.01663	exonic	nonsynonymous SNV	6	0.01066	cg06856840	1.648E-02	.	-6.95	
BPHL	chr6	+	rs2231365	0.01671	splicing	.	24	0.01671	cg22799902	6.218E-03	Island	-6.43	
ARHGAP10	chr4	+	rs144604709	0.0183	exonic	nonsynonymous SNV	0	0.0183	ch.4.2714611R	2.836E-02	.	-4.78	
ACTN3	chr11		rs201576110	0.02133	exonic	nonsynonymous SNV	32	0.02133	cg25117505	6.388E+08	Island	-8.39	
FGD6	chr12		rs143773760	0.02322	exonic	nonsynonymous SNV	9	0.02322	cg02688710	2.339E-02	Island	-6.09	Yes
WDR66	chr12	+	rs77422261	0.02336	exonic	nonsynonymous SNV	30	0.02336	cg21016266	1.331E-02	Island	-4.89	
RBM47	chr4	-	rs1688939	0.02571	intronic	.	.	0.02571	cg00802478	4.080E-02	.	-5.98	Yes
IQSEC1	chr3	-	rs144790333	0.02763	exonic	nonsynonymous SNV	23	0.02763	cg02559896	6.666E-03	Island	-5.14	
DLG1	chr3	-	rs141544348	0.03024	exonic	nonsynonymous SNV	35	6.01E-04	cg12594803	2.879E-02	.	-7.04	Yes
DUOX1	chr15		rs143304688	0.03047	exonic	stopgain	44	0.03047	cg10496082	1.526E-04	Island	-6.61	
RTDR1	chr22		rs3788337	0.03193	intronic	.	.	0.03193	cg21100191	3.904E-02	Island	-5.23	
CCR5,CCRL2	chr3	+	.	0.03194	intergenic	.	.	0.03194	cg10952220	1.002E-02	.	-7.39	
CCRL2	chr3	+	.	0.03355	downstream	.	.	0.03194	cg10952220	1.002E-02	.	-7.39	
PCDHA3	chr5		rs146951816	0.0354	exonic	nonsynonymous SNV	15	0.0354	cg02357321	3.348E-02	N_Shore	-6.43	
PARP11,CCND2	chr12	+	rs11063069	0.03845	intergenic	.	.	0.01055	cg05987650	8.273E-03	Island	-6.49	
C6orf70	chr6		rs140632188	0.04056	exonic	nonsynonymous SNV	23	0.04056	cg22807378	1.211E-02	Island	-7.15	
IGHMBP2	chr11		rs17612126	0.04126	exonic	nonsynonymous SNV	1	4.00E-03	cg26065952	2.314E-02	N_Shore	-5.70	Yes
TCTE3	chr6		rs116980543	0.04138	exonic	nonsynonymous SNV	10	0.04138	cg22807378	1.211E-02	Island	-7.15	
HERV-Fb1,PKNOX1	chr21	+	.	0.04328	intergenic	.	.	8.18E-03	cg04193427	4.013E-02	Island	-5.38	
FGFR4	chr5	+	.	0.04391	exonic	synonymous SNV	.	0.04391	cg21042539	4.943E-02	Island	-5.63	
CLRN2	chr4	+	rs201124485	0.04711	exonic	nonsynonymous SNV	21	0.04711	cg16760587	1.143E-03	S_Shelf	-6.06	
COL15A1	chr9	+	rs199906142	3.62E-03	exonic	nonsynonymous SNV	23	0.003618	cg18115656	3.656E-02	Island	5.37	
TNRC18	chr7	-	rs112785272	6.27E-03	exonic	nonsynonymous SNV	19	6.27E-03	cg10546562	1.010E-02	N_Shore	5.91	
SLC26A7	chr8	+	rs200788056	9.04E-03	exonic	nonsynonymous SNV	23	9.04E-03	cg25481252	2.582E-03	.	7.38	
DOCK8	chr9	+	rs77399114	9.95E-03	exonic	nonsynonymous SNV	9	9.95E-03	cg13876553	2.938E-02	.	5.41	
PCNX	chr14	+	rs200261097	0.01309	exonic	nonsynonymous SNV	32	0.01309	cg10066683	4.218E-02	.	5.34	Yes
PRICKLE2,ADAMTS9	chr3	-	rs7648640	0.01701	intergenic	.	.	0.01701	cg11044162	2.539E-02	N_Shore	4.75	
WDR35	chr2	-	rs148436608	0.02293	exonic	nonsynonymous SNV	16	0.02293	cg13734338	2.796E-02	.	6.00	Yes
SLC1A5	chr19	-	rs3027961	0.02329	exonic	nonsynonymous SNV	7	0.02329	cg08533710	1.634E-02	S_Shore	5.00	
ATXN7L1	chr7	-	rs10435352	0.03096	intronic	.	.	0.03096	cg05424831	3.142E-03	.	5.46	
MEGF6	chr1	-	rs61910697	0.03109	exonic	nonsynonymous SNV	20	0.03109	cg04391135	3.451E-03	Island	6.21	
DOCK8	chr9	+	rs12347078	0.03557	intronic	.	.	9.95E-03	cg13876553	2.938E-02	.	5.41	
PCNX	chr14	+	rs151313378	0.03622	exonic	nonsynonymous SNV	15	0.01309	cg10066683	4.218E-02	.	5.34	Yes
MEGF6	chr1	-	rs12075570	0.0367	exonic	nonsynonymous SNV	9	0.03109	cg04391135	3.451E-03	Island	6.21	
AGAP1	chr2	+	rs2292708	0.03677	exonic	synonymous SNV	.	0.03677	cg00834223	4.423E-03	.	6.19	
ARHGAP8,PRR5-ARH	chr22	+	rs55849456	0.03933	exonic	nonsynonymous SNV	25	0.03933	cg06647930	4.418E-02	S_Shelf	4.26	Yes
C1orf94	chr1	+	rs61741807	0.04033	exonic	nonsynonymous SNV	0	0.04033	cg15098077	1.363E-04	N_Shore	7.96	
PBX1,LMX1A	chr1	+	.	0.04041	intergenic	.	.	0.04041	cg20682146	4.200E-02	Island	6.22	
COL1A2	chr7	+	.	0.04129	exonic	synonymous SNV	.	0.04129	cg12563520	3.706E-02	.	6.05	
HS6ST3	chr13	+	rs4771948	0.04256	intronic	.	.	0.04256	cg09122588	4.527E-02	.	5.11	
ARHGAP8,PRR5-ARH	chr22	+	rs16992915	0.04662	exonic	nonsynonymous SNV	19	0.03933	cg06647930	4.418E-02	S_Shelf	4.26	Yes
ESYT2	chr7	-	rs2305475	0.04728	exonic	nonsynonymous SNV	23	0.04728	cg19584649	9.356E+09	.	6.66	Yes
AHRR	chr5	+	rs201569850	0.04754	exonic	nonsynonymous SNV	12	0.04754	cg16577724	3.796E-02	.	5.86	Yes
ESYT2	chr7	-	rs2305473	0.04755	exonic	nonsynonymous SNV	14	0.04728	cg19584649	9.356E+09	.	6.66	Yes
ESYT2	chr7	-	rs13233513	0.0479	exonic	nonsynonymous SNV	13	0.04728	cg19584649	9.356E+09	.	6.66	Yes

Table S3

Genesymbol	Chr	Strand	GWAS SNPs						DNA-methylation probes				Validated by gene-expression
			Id	*P*	Location	Mutation type	CADD score	min(*P*) for gene	Id	P_{BY}	Location	Tstat	
AP3M1	chr21			0.0121	intronic			0.0121	cg04760117	0.0486004	NaN	4.913713	
ZMIZ1/LOC283050	chr11		rs12571751	0.0357	intronic			0.0357	cg19168578	0.0486004	Island	-4.90911	
ELP2/SLC39A6	chr11	-	rs16967474	0.0322	exonic	nonsynonymous SNV	25.3	0.0322	cg12339809	0.0486004	Island	-5.07953	Yes

Table S4

		lists continues (1)		lists continues (2)		lists continues (3)	
Gene-symbol	Degree	Gene-symbol	Degree	Gene-symbol	Degree	Gene-symbol	Degree
THRAP3	33	*MDGA1*	11	*PACS2*	4	*COL15A1*	0
C5orf45	33	*SLC1A5*	11	*DUOX1*	4	*FRMD4A*	0
ANKRD17	32	*B3GALT6*	10	*EVI5L*	4	*MRPL21*	0
ATXN7L1	32	*C2orf63*	10	*PPP1R8*	3	*TSKU*	0
GPR176	32	*DOCK8*	10	*FYN*	3	*PTGER2*	0
IGHMBP2	31	*DCLK1*	10	*TATDN1*	3	*MPV17L*	0
PCNX	31	*LSM14A*	10	*UBE2D1*	3		
ADAR	30	*RBM47*	9	*WDR66*	3		
GAK	30	*CAMLG*	9	*PLEKHG3*	3		
ZNF544	30	*SSH3*	9	*TMC7*	3		
BOP1	29	*CMIP*	9	*DYNLL2*	3		
RTN1	29	*PALLD*	8	*SIGLEC5*	3		
POLR2A	29	*FAM35A*	8	*RABGGTB*	2		
SSTR2	29	*CLEC2B*	8	*CCNYL1*	2		
TNRC18	28	*VEZT*	8	*CMTM7*	2		
PKNOX1	28	*GPN3*	8	*AADAT*	2		
SDF4	27	*BTBD3*	8	*BPHL*	2		
RAB1A	26	*BCAR3*	7	*NEDD9*	2		
TRIM23	26	*ITPKB*	7	*KIAA1147*	2		
UNC45A	26	*RPIA*	7	*RPS13*	2		
GNL3	24	*LY96*	7	*PTPRJ*	2		
DIP2C	24	*GLUD1*	7	*FAH*	2		
ARHGEF3	23	*NINJ2*	7	*RNF220*	1		
PBRM1	22	*METTL7A*	7	*NOL10*	1		
ERC1	22	*TFDP1*	7	*LMAN2*	1		
RFWD3	22	*ZNF444*	7	*GMNN*	1		
UNK	22	*CCRL2*	6	*C6orf70*	1		
TGOLN2	21	*ADAMTS9*	6	*COL1A2*	1		
GGA1	21	*DLG1*	6	*KIAA1274*	1		
PTP4A2	20	*FGD4*	6	*POLR2G*	1		
ACTR10	18	*SAP18*	6	*RASSF8*	1		
FAM110B	17	*JMJD6*	6	*PRSS36*	1		
IQSEC1	16	*PIK3R3*	5	*AATF*	1		
FAM50B	16	*PBX1*	5	*MEGF6*	0		
ESYT2	15	*LRRC2*	5	*DNALI1*	0		
RBM14	15	*EHHADH*	5	*GSTM1*	0		
RAB6A	15	*FGFR4*	5	*OLFML2B*	0		
HPS6	14	*WIPF3*	5	*WDR35*	0		
GSDMD	13	*CDKN1C*	5	*AGAP1*	0		
MTRF1	13	*C12orf29*	5	*ARHGAP10*	0		
HP1BP3	12	*HAR1A*	5	*SAP30*	0		
C5orf44	12	*AKIRIN1*	4	*ARL15*	0		
UBTD2	12	*NPY1R*	4	*PEX7*	0		
FUCA2	12	*AHRR*	4	*TCTE3*	0		
PTPRN2	12	*RNASEH2C*	4	*ABHD11*	0		
MAP3K8	12	*ZDHHC24*	4	*HSPB1*	0		
CNKSR1	11	*CCND2*	4	*C8orf44*	0		
GRK6	11	*FGD6*	4	*NDUFB9*	0		

Table S5

Gene-symbol	GSE18632	GSE13162	GSE68605	GSE40438	Overlap with differential DNA-methylated genes in FTD-ALS
AATF	TRUE				TRUE
ABHD11					TRUE
ADAMTS9					TRUE
ADAR					TRUE
AGAP1					TRUE
AHRR	TRUE				TRUE
AKIRIN1	TRUE				TRUE
ANKRD17	TRUE				TRUE
APOB		TRUE			TRUE
ARHGEF3	TRUE				TRUE
ARL15	TRUE				TRUE
BCAR3					TRUE
BOP1					TRUE
BTBD3					TRUE
C1ORF61	TRUE				TRUE
CCND2	TRUE	TRUE			TRUE
CCNYL1					TRUE
CCRL2					TRUE
CDKN1C		TRUE			TRUE
CLEC2B					TRUE
CMIP					TRUE
CNKSR1		TRUE			TRUE
COL15A1					TRUE
COL1A2					TRUE
DCLK1					TRUE
DIP2C					TRUE
DLG1		TRUE			TRUE
DUOX1					TRUE
DYNLL2	TRUE				TRUE
ERC1	TRUE				TRUE
ESYT2	TRUE				TRUE
EVI5L					TRUE
FAH					TRUE
FAM110B					TRUE
FAM24B	TRUE				TRUE
FGD6	TRUE				TRUE
FGFR4					TRUE
FYN		TRUE			TRUE
GABRG2					TRUE
GAK	TRUE				TRUE
GLUD1					TRUE
GNL3					TRUE
GPR176					TRUE
GRK6	TRUE				TRUE
GSDMD					TRUE
GSTM1					TRUE

HOXA4		TRUE			TRUE
HP1BP3					TRUE
HSPB1	TRUE				TRUE
IGHMBP2		TRUE			TRUE
ITPKB		TRUE			TRUE
JPH3	TRUE				TRUE
KCNQ2		TRUE			TRUE
KIAA1147	TRUE				TRUE
LAMP3	TRUE				TRUE
LMAN2		TRUE			TRUE
LRRC2					TRUE
LSM14A	TRUE				TRUE
LY96					TRUE
MDGA1					TRUE
MEGF6					TRUE
MEOX1		TRUE			TRUE
METTL7A	TRUE	TRUE			TRUE
MPV17L					TRUE
MRPL21	TRUE				TRUE
MYF6					TRUE
NDUFB9					TRUE
NINJ2		TRUE			TRUE
OLFML2B					TRUE
PACS2					TRUE
PALLD	TRUE	TRUE			TRUE
PBRM1	TRUE				TRUE
PBX1					TRUE
PCDHA2					TRUE
PCDHA3					TRUE
PCDHA5					TRUE
PCNX	TRUE	TRUE			TRUE
PEX7					TRUE
PIK3R3	TRUE				TRUE
PKNOX1		TRUE			TRUE
PLEKHG3		TRUE			TRUE
POLR2A					TRUE
POLR2G					TRUE
PRR5-ARHGAP8	TRUE				TRUE
PTGER2					TRUE
PTP4A2	TRUE	TRUE			TRUE
PTPRJ	TRUE				TRUE
PTPRN2		TRUE			TRUE
RAB1A	TRUE				TRUE
RAB6A		TRUE			TRUE
RABGGTB					TRUE
RASSF8					TRUE
RBM14					TRUE
RBM47	TRUE				TRUE
RPIA					TRUE
RPS13		TRUE			TRUE

RTN1					TRUE
SAP18					TRUE
SAP30		TRUE			TRUE
SHANK1		TRUE			TRUE
SLC1A5					TRUE
SLC26A7					TRUE
SSH3					TRUE
SSTR2		TRUE			TRUE
TCTE3					TRUE
TFDP1	TRUE				TRUE
TGOLN2					TRUE
THRAP3					TRUE
TLX3		TRUE			TRUE
TNRC18					TRUE
TRIM23	TRUE				TRUE
TRIM61					TRUE
UBE2D1		TRUE			TRUE
UBTD2	TRUE				TRUE
UNK					TRUE
VEZT		TRUE			TRUE
WDR35	TRUE				TRUE
WIPF3	TRUE				TRUE
ZNF444		TRUE			TRUE

Table S6

Data set	GEO accesion nr.	#Genes with $P_{BH} \leq 0.05$	#Genes in overlap with FTD-ALS	*P*	#Genes in overlap with FTD	*P*
1	GSE18632	4407	36	0.0276*	1	0.7666
2	GSE13162	2572	29	0.0445*	0	1
3	GSE68605	7	0	1	0	1
4	GSE40438	0	-	-	-	-
4						

Table S7

Data used in our study	Genesymbol	Probe id	chr	Location (range)	Marie-Laure Caillet-Boudin et al, 2015	*Daniela Galimberti et al, 2013*	Russ, J. et al, 2012
					MAPT - extended CpG regions	*GRN - (entire open reading frame)*	*C9orf72 - (2KB upstream promoter region)*
					44060543-44061296 (CpG_{21}) 43972053-44039685 (CpG_{27}) 43971747-43972052 (CpG_{300})	43971748-44105699	27573864-27575864
Li, Y et al	*Mapt*	cg05533539	chr17	44104521-44104571			
Li, Y et al	*Grn*	cg19107120	chr17	42430376-42430426			
Li, Y et al	*Grn*	cg11999384	chr17	42422811-42422861			
Li, Y et al	*Grn*	cg12837296	chr17	42426483-42426533			
Li, Y et al	*Grn*	cg20679560	chr17	42423025-42423075			
Li, Y et al	*Grn*	cg23570245	chr17	42426011-42426061			
Li, Y et al	*Grn*	cg24420717	chr17	42426506-42426556			
Li, Y et al	*Grn*	cg17714154	chr17	42422247-42422297			
Li, Y et al	*Grn*	cg18872749	chr17	42421993-42422043			
Li, Y et al	*Grn*	cg01146514	chr17	42422409-42422459			
Li, Y et al	*Grn*	cg04269641	chr17	42422442-42422492			
Li, Y et al	*Grn*	cg07777378	chr17	42422412-42422462			
Li, Y et al	*Grn*	cg17101358	chr17	42422688-42422738			
Li, Y et al	*C9orf72*	cg13958452	chr9	27571484-27571534			
Li, Y et al	*C9orf72*	cg05990720	chr9	27573650-27573700			
Li, Y et al	*C9orf72*	cg11613875	chr9	27573889-27573939			X
Li, Y et al	*C9orf72*	cg14363787	chr9	27573986-27574036			X

Table S8

Padjusted	Probe id	Genesymbol
0.0007	cg16316507	tpk1
0.0010	cg21369679	dnaja2
0.0015	cg08312369	cdkn2d
0.0015	cg19285752	rai1
0.0018	cg12339809	elp2;slc39a6
0.0037	cg06358566	rpia
0.0047	cg04760117	ap3m1
0.0062	cg26771998	bms1p4
0.0083	cg14948912	gatad2a
0.0083	cg01533966	lrrc8d
0.0087	cg11759644	rnf138
0.0098	cg18562663	bcl6
0.0128	cg19576806	dhrs12
0.0135	cg03431064	tubb2c
0.0156	cg11457817	stc2
0.0180	cg26840318	irf2bp2
0.0195	cg03511282	ntan1
0.0204	cg01404182	c6orf120;wdr27
0.0206	cg17222196	efna3
0.0208	cg03159329	stk39
0.0213	cg06951969	smad6
0.0227	cg14325160	rad23b
0.0246	cg08090063	plcd1
0.0262	cg11952315	nags
0.0268	cg11342046	ing5
0.0275	cg02988046	egln1
0.0285	cg11760861	adat3;scamp4
0.0308	cg02504384	tsc2
0.0327	cg12588699	c1orf96
0.0358	cg19168578	loc283050;zmiz1
0.0411	cg19791630	vav1
0.0437	cg13714039	znf14
0.0446	cg05554039	map6
0.0457	cg08797483	calcr

APPENDIX A

Acknowledgments

Intramural funding from the National Institute of Neurological Disorders and Stroke (NINDS) and National Institute on Aging (NIA), the Wellcome/MRC Centre on Parkinson's disease, Alzheimer's Research UK (ARUK, Grant ARUK-PG2012-18) and by the office of the Dean of the School of Medicine, Department of Internal Medicine, at Texas Tech University Health Sciences Center.

We thank Mike Hubank and Kerra Pearce at the Genomic core facility at the Institute of Child Health (ICH), University College of London (UCL), for assisting RF in performing Illumina genotyping experiments (FTD-GWAS genotyping). This study utilized the high-performance computational capabilities of the Biowulf Linux cluster at the National Institutes of Health, Bethesda, Md. (http://biowulf.nih.gov). North American Brain Expression Consortium (NABEC) - The work performed by the North American Brain Expression Consortium (NABEC) was supported in part by the Intramural Research Program of the National Institute on Aging, National Institutes of Health, part of the US Department of Health and Human Services; project number ZIA AG000932-04. In addition this work was supported by a Research Grant from the Department of Defense, W81XWH-09-2-0128. UK Brain Expression Consortium (UKBEC) - This work performed by the UK Brain Expression Consortium (UKBEC) was supported by the MRC through the MRC Sudden Death Brain Bank (C.S.), by a Project Grant (G0901254 to J.H. and M.W.) and by a Fellowship award (G0802462 to M.R.). D.T. was supported by the King Faisal Specialist Hospital and Research Centre, Saudi Arabia. Computing facilities used at King's College London were supported by the National Institute for Health Research (NIHR) Biomedical Research Centre based at Guy's and St Thomas' NHS Foundation Trust and King's College London. We would like to thank AROS Applied Biotechnology AS company laboratories and Affymetrix for their valuable input. RF's work is supported by Alzheimer's Society (grant number 284), UK; JBJK was supported by the National Health and Medical Resarch Council (NHMRC) Australia, Project Grants 510217 and 1005769; CDS was supported by NHMRC Project Grants 630428 and 1005769; PRS was supported by NHMRC Project Grants 510217 and 1005769 and acknowledges that DNA samples were prepared by Genetic Repositories Australia, supported by NHMRC Enabling Grant 401184; GMH was supported by NHMRC Research Fellowship 630434, Project Grant 1029538, Program Grant 1037746; JRH was supported by the Australian Research Council Federation Fellowship, NHMRC Project Grant 1029538, NHMRC Program Grant 1037746; OP was supported by NHMRC Career Development Fellowship 1022684, Project Grant 1003139. IH, AR and MB acknowledge the patients and controls who participated in this project and the Trinitat Port-Carbó and her family who are supporting Fundació ACE research programs. CC was supported by Grant P30-NS069329-01 and acknowledges that the recruitment and clinical characterization of research participants at Washington University were supported by NIH P50 AG05681, P01 AG03991, and P01 AG026276. LB and GB were supported by the Ricerca Corrente, Italian Ministry of Health; RG was supported by Fondazione CARIPLO 2009-2633, Ricerca Corrente, Italian

Ministry of Health; GF was supported by Fondazione CARIPLO 2009-2633. ES was supported by the Italian Ministry of Health; CF was supported by Fondazione Cariplo; MS was supported from the Italian Ministry of Health (Ricerca Corrente); MLW was supported by Government funding of clinical research within NHS Sweden (ALF); KN was supported by Thure Carlsson Foundation; CN was supported by Swedish Alzheimer Fund. IRAM and GYRH were supported by CIHR (grant 74580) PARF (grant C06-01). JG was supported by the NINDS intramural research funds for FTD research. CMM was supported by Medical Research Council UK, Brains for Dementia Research, Alzheimer's Society, Alzheimer's Research UK, National Institutes for Health Research, Department of Health, Yvonne Mairy Bequest and acknowledges that tissue made available for this study was provided by the Newcastle Brain Tissue Resource, which was funded in part by grants G0400074 and G1100540 from the UK MRC, the Alzheimer's Research Trust and Alzheimer's Society through the Brains for Dementia Research Initiative and an NIHR Biomedical Research Centre Grant in Ageing and Health, and NIHR Biomedical Research Unit in Lewy Body Disorders. CMM was supported by the UK Department of Health and Medical Research Council and the Research was supported by the National Institute for Health Research Newcastle Biomedical Research Centre based at Newcastle Hospitals Foundation Trust and Newcastle University and acknowledges that the views expressed are those of the authors and not necessarily those of the NHS, the NIHR or the Department of Health; JA was supported by MRC, Dunhill Medical Trust, Alzheimer's Research UK; TDG was supported by Wellcome Trust Senior Clinical Fellow; IGM was supported by NIHR Biomedical Research Centre and Unit on Ageing Grants and acknowledges the National Institute for Health Research Newcastle Biomedical Research Centre based at Newcastle Hospitals Foundation Trust and Newcastle University. The views expressed are those of the author(s) and not necessarily those of the NHS, the NIHR or the Department of Health; AJT was supported by Medical Research Council, Alzheimer's Society, Alzheimer's Research UK, National Institutes for Health Research. EJ was supported by NIHR, Newcastle Biomedical Research Centre. PP, CR, SOC and EA were supported partially by FIMA (Foundation for Applied Medical Research); PP acknowledges Manuel Seijo-Martínez (Department of Neurology, Hospital do Salnés, Pontevedra, Spain), Ramon Rene, Jordi Gascon and Jaume Campdelacreu (Department of Neurology, Hospital de Bellvitge, Barcelona, Spain) for providing FTD DNA samples. RP, JDS, PA and AK were supported by German Federal Ministry of Education and Research (BMBF; grant number FKZ 01GI1007A – German FTLD consortium). IR was supported by Ministero dell'Istruzione, dell'Università e della Ricerca (MIUR) of Italy. PStGH was supported by the Canadian Institutes of Health Research, Wellcome Trust, Ontario Research Fund. FT was supported by the Italian Ministry of Health (ricerca corrente) and MIUR grant RBAP11FRE9; GR and GG were supported by the Italian Ministry of Health (ricerca corrente). JBR was supported by Camrbidge NIHR Biomedical Research Centre and Wellcome Trust (088324). JU, JC, SM were supported by the MRC Prion Unit core funding and acknowledge MRC UK, UCLH Biomedical Research Centre, Queen Square Dementia BRU; SM acknowledges the work of John Beck, Tracy Campbell, Gary Adamson, Ron Druyeh, Jessica Lowe, Mark Poulter. AD acknowledges the work of Benedikt Bader and of Manuela Neumann, Sigrun Roeber, Thomas Arzberger and Hans Kretzschmar†; VMVD and JQT were supported by Grants AG032953, AG017586 and AG010124; MG was supported by Grants AG032953, AG017586, AG010124 and NS044266; VMVD acknowledges EunRan Suh, PhD for assistance with sample handling and Elisabeth

McCarty-Wood for help in selection of cases; JQT acknowledges Terry Schuck, John Robinson and Kevin Raible for assistance with neuropathological evaluation of cases. CVB and the Antwerp site were in part funded by the MetLife Foundation for Medical Research Award (to CVB), the Belgian Science Policy Office (BELSPO) Interuniversity Attraction Poles program; the Alzheimer Research Foundation (SAO-FRA); the Medical Foundation Queen Elisabeth (GSKE); the Flemish Government initiated Methusalem Excellence Program (to CVB); the Research Foundation Flanders (FWO) and the University of Antwerp Research Fund.. CVB, MC and JvdZ acknowledge the neurologists S Engelborghs, PP De Deyn, A Sieben, R Vandenberghe and the neuropathologist JJ Martin for the clinical and pathological diagnoses. CVB, MC and JvdZ further thank the personnel of the Genetic Service Facility of the VIB Department of Molecular Genetics (http://www.vibgeneticservicefacility.be) and the Antwerp Biobank of the Institute Born-Bunge for their expert support. IL and AB were supported by the program “Investissements d’avenir” ANR-10-IAIHU-06 and acknowledges the contribution of The French research network on FTLD/FTLD-ALS for the contribution in samples collection. BN is founded by Fondazione Cassa di Risparmio di Pistoia e Pescia (grant 2014.0365), SS is founded by the Cassa di Risparmio di Firenze (grant 2014.0310) and a grant from Ministry of Health n° RF-2010-2319722. JEN was supported by the Novo Nordisk Foundation, Denmark. MR was supported by the German National Genome Network (NGFN); German Ministry for Education and Research Grant Number 01GS0465. JDR, MNR, NCF and JDW were supported by an MRC programme grant and the Dementia Platform UK, the NIHR Queen Square Dementia Biomedical Research Unit (BRU) and the Leonard Wolfson Experimental Neurology Centre. MGS was supported by MRC grant n G0301152, Cambridge Biomedical Research Centre and acknowledges Mrs K Westmore for extracting DNA. HM was supported by the Motor Neuron Disease Association (Grant 6057). RR was supported by P50 AG016574, R01 NS080882, R01 NS065782, P50 NS72187 and the Consortium for Frontotemporal Dementia; DWD was supported by P50NS072187, P50AG016574, State of Florida Alzheimer Disease Initiative, & CurePSP, Inc.; NRGR, JEP, RCP, DK, BFB were supported by P50 AG016574; KAJ was supported by R01 AG037491; WWS was supported by NIH AG023501, AG019724, Consortium for Frontotemporal Dementia Research; BLM was supported by P50AG023501, P01AG019724, Consortium for FTD Research; HR was supported by AG032306. JCvS was supported by Stichting Dioraphte Foundation (11 02 03 00), Nuts Ohra Foundation (0801-69), Hersenstichting Nederland (BG 2010-02) and Alzheimer Nederland. CG and HHC acknowledge families, patients, clinicians including Dr Inger Nennesmo and Dr Vesna Jelic, Professor Laura Fratiglioni for control samples and Jenny Björkström, Håkan Thonberg, Charlotte Forsell, Anna-Karin Lindström and Lena Lilius for sample handling. CG was supported by Swedish Brain Power (SBP), the Strategic Research Programme in Neuroscience at Karolinska Institutet (StratNeuro), the regional agreement on medical training and clinical research (ALF) between Stockholm County Council and Karolinska Institutet, Swedish Alzheimer Foundation, Swedish Research Council, Karolinska Institutet PhD-student funding, King Gustaf V and Queen Victoria’s Free Mason Foundation. FP, AR, VD and FL acknowledge Labex DISTALZ. RF acknowledges the help and support of Mrs. June Howard at the Texas Tech University Health Sciences Center Office of Sponsored Programs for tremendous help in managing Material Transfer Agreement at TTUHSC.

APPENDIX B

IFGC members and affiliations

Raffaele Ferrari (UCL, Department of Molecular Neuroscience, Russell Square House, 9-12 Russell Square House, London, WC1B 5EH), Dena G Hernandez (Laboratory of Neurogenetics, National Institute on Aging, National Institutes of Health, Building 35, Room 1A215, 35 Convent Drive, Bethesda, MD 20892, USA; Reta Lila Weston Research Laboratories, Department of Molecular Neuroscience, UCL Institute of Neurology, London WC1N 3BG, UK), Michael A Nalls (Laboratory of Neurogenetics, National Institute on Aging, National Institutes of Health Building 35, Room 1A215, 35 Convent Drive, Bethesda, MD 20892, USA), Jonathan D Rohrer (Reta Lila Weston Research Laboratories, Department of Molecular Neuroscience, UCL Institute of Neurology, Queen Square, London WC1N 3BG, UK; Dementia Research Centre, Department of Neurodegenerative Disease, UCL Institute of Neurology), Adaikalavan Ramasamy (Reta Lila Weston Research Laboratories, Department of Molecular Neuroscience, UCL Institute of Neurology, London WC1N 3BG, UK; Department of Medical and Molecular Genetics, King's College London Tower Wing, Guy's Hospital, London SE1 9RT, UK; The Jenner Institute, University of Oxford, Roosevelt Drive, Oxford OX3 7BQ, UK), John BJ Kwok (Neuroscience Research Australia, Sydney, NSW 2031, Australia; School of Medical Sciences, University of New South Wales, Sydney, NSW 2052, Australia), Carol Dobson-Stone (Neuroscience Research Australia, Sydney, NSW 2031, Australia; School of Medical Sciences, University of New South Wales, Sydney, NSW 2052, Australia), William S Brooks Neuroscience Research Australia, Sydney, NSW 2031, Australia; Prince of Wales Clinical School, University of New South Wales, Sydney, NSW 2052, Australia), Peter R Schofield (Neuroscience Research Australia, Sydney, NSW 2031, Australia; School of Medical Sciences, University of New South Wales, Sydney, NSW 2052, Australia), Glenda M Halliday (Neuroscience Research Australia, Sydney, NSW 2031, Australia; School of Medical Sciences, University of New South Wales, Sydney, NSW 2052, Australia), John R Hodges (Neuroscience Research Australia, Sydney, NSW 2031, Australia; School of Medical Sciences, University of New South Wales, Sydney, NSW 2052, Australia), Olivier Piguet (Neuroscience Research Australia, Sydney, NSW 2031, Australia; School of Medical Sciences, University of New South Wales, Sydney, NSW 2052, Australia), Lauren Bartley (Neuroscience Research Australia, Sydney, NSW 2031, Australia), Elizabeth Thompson (South Australian Clinical Genetics Service, SA Pathology (at Women's and Children's Hospital), North Adelaide, SA 5006, Australia; Department of Paediatrics, University of Adelaide, Adelaide, SA 5000, Australia), Eric Haan (South Australian Clinical Genetics Service, SA Pathology (at Women's and Children's Hospital), North Adelaide, SA 5006, Australia; Department of Paediatrics, University of Adelaide, Adelaide, SA 5000, Australia), Isabel Hernández (Research Center and Memory Clinic of Fundació ACE, Institut Català de Neurociències Aplicades, Barcelona, Spain), Agustín Ruiz (Research Center and Memory Clinic of Fundació ACE, Institut Català de Neurociències Aplicades, Barcelona, Spain), Mercè Boada (Research Center and Memory Clinic of Fundació ACE, Institut Català de Neurociències Aplicades, Barcelona, Spain), Barbara Borroni (Neurology Clinic, University of Brescia, Brescia, Italy), Alessandro Padovani (Neurology Clinic, University of Brescia, Brescia, Italy), Carlos Cruchaga (Department of Psychiatry, Washington University, St. Louis, MO, USA; Hope Center, Washington University School of Medicine, St. Louis, MO, USA), Nigel J Cairns (Hope Center, Washington University School of Medicine, St. Louis, MO, USA; Department of Pathology and Immunology, Washington University, St. Louis, MO, USA), Luisa Benussi (Molecular Markers Laboratory, IRCCS Istituto Centro San Giovanni di Dio

Fatebenefratelli,Brescia, Italy), Giuliano Binetti (MAC Memory Clinic, IRCCS Istituto Centro San Giovanni di Dio Fatebenefratelli, Brescia, Italy), Roberta Ghidoni (Molecular Markers Laboratory, IRCCS Istituto Centro San Giovanni di Dio Fatebenefratelli, Brescia, Italy), Gianluigi Forloni (Biology of Neurodegenerative Disorders, IRCCS Istituto di Ricerche Farmacologiche, "Mario Negri", Milano, Italy), Diego Albani (Biology of Neurodegenerative Disorders, IRCCS Istituto di Ricerche Farmacologiche "Mario Negri", Milano, Italy), Daniela Galimberti (University of Milan, Milan, Italy; Fondazione Cà Granda, IRCCS Ospedale Maggiore Policlinico, via F. Sforza 35, 20122, Milan, Italy), Chiara Fenoglio (University of Milan, Milan, Italy; Fondazione Cà Granda, IRCCS Ospedale Maggiore Policlinico, via F. Sforza 35, 20122, Milan, Italy), Maria Serpente (University of Milan, Milan, Italy; Fondazione Cà Granda, IRCCS Ospedale Maggiore Policlinico, via F. Sforza 35, 20122, Milan, Italy), Elio Scarpini (University of Milan, Milan, Italy; Fondazione Cà Granda, IRCCS Ospedale Maggiore Policlinico, via F. Sforza 35, 20122, Milan, Italy), Jordi Clarimón (Memory Unit, Neurology Department and Sant Pau Biomedical Research Institute, Hospital de la Santa Creu i Sant Pau, Universitat Autònoma de Barcelona, Barcelona, Spain; Center for Networker Biomedical Research in Neurodegenerative Diseases (CIBERNED), Madrid, Spain), Alberto Lleó (Memory Unit, Neurology Department and Sant Pau Biomedical Research Institute, Hospital de la Santa Creu i Sant Pau, Universitat Autònoma de Barcelona, Barcelona, Spain; Center for Networker Biomedical Research in Neurodegenerative Diseases (CIBERNED), Madrid, Spain), Rafael Blesa (Memory Unit, Neurology Department and Sant Pau Biomedical Research Institute, Hospital de la Santa Creu i Sant Pau, Universitat Autònoma de Barcelona, Barcelona, Spain; Center for Networker Biomedical Research in Neurodegenerative Diseases (CIBERNED), Madrid, Spain); Maria Landqvist Waldö (Unit of Geriatric Psychiatry, Department of Clinical Sciences, Lund University, Lund, Sweden), Karin Nilsson (Unit of Geriatric Psychiatry, Department of Clinical Sciences, Lund University, Lund, Sweden), Christer Nilsson (Clinical Memory Research Unit, Department of Clinical Sciences, Lund University, Lund, Sweden), Ian RA Mackenzie (Department of Pathology and Laboratory Medicine, University of British Columbia, Vancouver, Canada), Ging-Yuek R Hsiung (Division of Neurology, University of British Columbia, Vancouver, Canada), David MA Mann (Institute of Brain, Behaviour and Mental Health, University of Manchester, Salford Royal Hospital, Stott Lane, Salford, M6 8HD, UK), Jordan Grafman (Rehabilitation Institute of Chicago, Departments of Physical Medicine and Rehabilitation, Psychiatry, and Cognitive Neurology & Alzheimer's Disease Center; Feinberg School of Medicine, Northwestern University, Chicago, USA; Department of Psychology, Weinberg College of Arts and Sciences, Northwestern University, , Chicago, USA), Christopher M Morris (Newcastle Brain Tissue Resource, Institute for Ageing, Newcastle University, NE4 5PL, Newcastle upon Tyne, UK; Newcastle University, Institute of Neuroscience and Institute for Ageing, Campus for Ageing and Vitality, NE4 5PL, Newcastle upon Tyne, UK; Institute of Neuroscience, Newcastle University Medical School, Framlington Place NE2 4HH, Newcastle upon Tyne, UK), Johannes Attems (Newcastle University, Institute of Neuroscience and Institute for Ageing, Campus for Ageing and Vitality, NE4 5PL, Newcastle upon Tyne, UK; Timothy D Griffiths (Institute of Neuroscience, Newcastle University Medical School, Framlington Place, NE2 4HH, Newcastle upon Tyne, UK), Ian G McKeith (Newcastle University, Institute of Neuroscience and Institute for Ageing, Campus for Ageing and Vitality, Newcastle University, NE4 5PL, Newcastle upon Tyne, UK), Alan J Thomas (Newcastle University, Institute of Neuroscience and Institute for Ageing, Campus for Ageing and Vitality, NE4 5PL, Newcastle upon Tyne, UK), Pietro Pietrini (IMT School for Advanced Studies, Lucca, Lucca, Italy), Edward D Huey (Taub Institute, Departments of Psychiatry and Neurology, Columbia University, 630 West 168th Street New York, NY 10032), Eric M Wassermann (Behavioral Neurology Unit, National Insititute of Neurological Disorders and Stroke, National Insititutes of Health, 10 CENTER DR MSC 1440, Bethesda, MD 20892-1440), Atik Baborie (Department of Laboratory Medicine & Pathology, Walter Mackenzie Health Sciences Centre, 8440 - 112 St, University of Alberta Edmonton, Alberta T6G 2B7, Canada), Evelyn Jaros

(Newcastle University, Institute for Ageing and Health, Campus for Ageing and Vitality, NE4 5PL, Newcastle upon Tyne, UK), Michael C Tierney (Behavioral Neurology Unit, National Insititute of Neurological Disorders and Stroke, National Insititutes of Health, 10 CENTER DR MSC 1440, Bethesda, MD 20892-1440), Pau Pastor (Center for Networker Biomedical Research in Neurodegenerative Diseases (CIBERNED), Madrid, Spain; Neurogenetics Laboratory, Division of Neurosciences, Center for Applied Medical Research, Universidad de Navarra, Pamplona, Spain; Department of Neurology, Clínica Universidad de Navarra, University of Navarra School of Medicine, Pamplona, Spain), Cristina Razquin (Neurogenetics Laboratory, Division of Neurosciences, Center for Applied Medical Research, Universidad de Navarra, Pamplona, Spain), Sara Ortega-Cubero (Center for Networker Biomedical Research in Neurodegenerative Diseases (CIBERNED), Madrid, Spain; Neurogenetics Laboratory, Division of Neurosciences, Center for Applied Medical Research, Universidad de Navarra, Pamplona, Spain), Elena Alonso (Neurogenetics Laboratory, Division of Neurosciences, Center for Applied Medical Research, Universidad de Navarra, Pamplona, Spain), Robert Perneczky (Neuroepidemiology and Ageing Research Unit, School of Public Health, Faculty of Medicine, The Imperial College of Science, Technology and Medicine, London W6 8RP, UK; West London Cognitive Disorders Treatment and Research Unit, West London Mental Health Trust, London TW8 8 DS, UK; Department of Psychiatry and Psychotherapy, Technische Universität München, Munich, 81675 Germany), Janine Diehl-Schmid (Department of Psychiatry and Psychotherapy, Technische Universität München, Munich, 81675 Germany), Panagiotis Alexopoulos (Department of Psychiatry and Psychotherapy, Technische Universität München, Munich, 81675 Germany), Alexander Kurz (Department of Psychiatry and Psychotherapy, Technische Universität München, Munich, 81675 Germany), Innocenzo Rainero (Neurology I, Department of Neuroscience, University of Torino, Italy, A.O. Città della Salute e della Scienza di Torino, Torino, Italy), Elisa Rubino (Neurology I, Department of Neuroscience, University of Torino, Italy, A.O. Città della Salute e della Scienza di Torino, Torino, Italy), Lorenzo Pinessi (Neurology I, Department of Neuroscience, University of Torino, Italy, A.O. Città della Salute e della Scienza di Torino, Torino, Italy), Ekaterina Rogaeva (Tanz Centre for Research in Neurodegenerative Diseases, University of Toronto 60 Leonard Street, Toronto, Ontario, Canada, M5T 2S8), Peter St George-Hyslop(Tanz Centre for Research in Neurodegenerative Diseases, University of Toronto, 60 Leonard Street, Toronto, Ontario, Canada, M5T 2S8; Cambridge Institute for Medical Research, and the Department of Clinical Neurosciences, University of Cambridge, Hills Road, Cambridge, UK CB2 0XY), Giacomina Rossi (Division of Neurology V and Neuropathology, Fondazione IRCCS Istituto Neurologico Carlo Besta, 20133 Milano, Italy), Fabrizio Tagliavini (Division of Neurology V and Neuropathology, Fondazione IRCCS Istituto Neurologico Carlo Besta, 20133 Milano, Italy), Giorgio Giaccone (Division of Neurology V and Neuropathology, Fondazione IRCCS Istituto Neurologico Carlo Besta, 20133 Milano, Italy), James B Rowe Cambridge University Department of Clinical Neurosciences, Cambridge, CB2 0SZ, UK; MRC Cognition and Brain Sciences Unit, Cambridge, CB2 7EF, UK; Behavioural and Clinical Neuroscience Institute, Cambridge, CB2 3EB, UK), Johannes CM Schlachetzki (University of California San Diego, Department of Cellular & Molecular Medicine, 9500 Gilman Drive, La Jolla, CA, 92093), James Uphill (MRC Prion Unit, Department of Neurodegenerative Disease, UCL Institute of Neurology, Queen Square House, Queen Square, London, WC1N 3BG), John Collinge (MRC Prion Unit, Department of Neurodegenerative Disease, UCL Institute of Neurology Queen Square House, Queen Square, London, WC1N 3BG), Simon Mead (MRC Prion Unit, Department of Neurodegenerative Disease, UCL Institute of Neurology, Queen Square House, Queen Square, London, WC1N 3BG), Adrian Danek (Neurologische Klinik und Poliklinik, Ludwig-Maximilians-Universität, Munich, Germany, German Center for Neurodegenerative Diseases (DZNE)), Vivianna M Van Deerlin (University of Pennsylvania Perelman School of Medicine, Department of Pathology and Laboratory Medicine, Philadelphia, PA, USA), Murray Grossman (University of Pennsylvania Perelman School of

Medicine, Department of Neurology and Penn Frontotemporal Degeneration Center, Philadelphia, PA, USA), John Q Trojanowski (University of Pennsylvania Perelman School of Medicine, Department of Pathology and Laboratory Medicine, Philadelphia, PA, USA), Julie van der Zee (Neurodegenerative Brain Diseases group, Department of Molecular Genetics, VIB, Antwerp, Belgium; Laboratory of Neurogenetics, Institute Born-Bunge, University of Antwerp, Antwerp, Belgium), Marc Cruts (Neurodegenerative Brain Diseases group, Department of Molecular Genetics, VIB, Antwerp, Belgium; Laboratory of Neurogenetics, Institute Born-Bunge, University of Antwerp, Antwerp, Belgium), Christine Van Broeckhoven (Neurodegenerative Brain Diseases group, Department of Molecular Genetics, VIB, Antwerp, Belgium; Laboratory of Neurogenetics, Institute Born-Bunge, University of Antwerp, Antwerp, Belgium), Stefano F Cappa (Neurorehabilitation Unit, Dept. Of Clinical Neuroscience, Vita-Salute University and San Raffaele Scientific Institute, Milan, Italy), Isabelle Leber (Inserm, UMR_S975, CRICM; UPMC Univ Paris 06, UMR_S975; CNRS UMR 7225, F-75013, Paris, France; AP-HP, Hôpital de la Salpêtrière, Département de neurologie-centre de références des démences rares, F-75013, Paris, France), Didier Hannequin (Service de Neurologie, Inserm U1079, CNR-MAJ, Rouen University Hospital, Rouen, France), Véronique Golfier (Service de neurologie, CH Saint Brieuc, France), Martine Vercelletto (Service de neurologie, CHU Nantes, France), Alexis Brice (Inserm, UMR_S975, CRICM; UPMC Univ Paris 06, UMR_S975; CNRS UMR 7225, F-75013, Paris, France; AP-HP, Hôpital de la Salpêtrière, Département de neurologie-centre de références des démences rares, F-75013, Paris, France), Benedetta Nacmias (Department of Neurosciences, Psychology, Drug Research and Child Health (NEUROFARBA) University of Florence, Florence, Italy), Sandro Sorbi (Department of Neurosciences, Psychology, Drug Research and Child Health (NEUROFARBA), University of Florence and IRCCS "Don Carlo Gnocchi" Firenze, Florence, Italy), Silvia Bagnoli (Department of Neurosciences, Psychology, Drug Research and Child Health (NEUROFARBA), University of Florence, Florence, Italy), Irene Piaceri (Department of Neurosciences, Psychology, Drug Research and Child Health (NEUROFARBA), University of Florence, Florence, Italy), Jørgen E Nielsen (Danish Dementia Research Centre, Neurogenetics Clinic, Department of Neurology, Rigshospitalet, Copenhagen University Hospital, Copenhagen, Denmark; Department of Cellular and Molecular Medicine, Section of Neurogenetics, The Panum Institute, University of Copenhagen, Copenhagen, Denmark), Lena E Hjermind (Danish Dementia Research Centre, Neurogenetics Clinic, Department of Neurology, Rigshospitalet, Copenhagen University Hospital, Copenhagen, Denmark; Department of Cellular and Molecular Medicine, Section of Neurogenetics, The Panum Institute, University of Copenhagen, Copenhagen, Denmark), Matthias Riemenschneider (Saarland University Hospital, Department for Psychiatry & Psychotherapy, Kirrberger Str.1, Bld.90, 66421 Homburg/Saar, Germany; Saarland University, Laboratory for Neurogenetics, Kirrberger Str.1, Bld.90, 66421 Homburg/Saar, Germany), Manuel Mayhaus (Saarland University, Laboratory for Neurogenetics, Kirrberger Str.1, Bld.90, 66421 Homburg/Saar, Germany), Bernd Ibach (University Regensburg, Department of Psychiatry, Psychotherapy and Psychosomatics, Universitätsstr. 84, 93053 Regensburg, Germany), Gilles Gasparoni (Saarland University, Laboratory for Neurogenetics, Kirrberger Str.1, Bld.90, 66421 Homburg/Saar, Germany), Sabrina Pichler (Saarland University, Laboratory for Neurogenetics, Kirrberger Str.1, Bld.90, 66421 Homburg/Saar, Germany), Wei Gu (Saarland University, Laboratory for Neurogenetics, Kirrberger Str.1, Bld.90, 66421 Homburg/Saar, Germany; Luxembourg Centre For Systems Biomedicine (LCSB), University of Luxembourg 7, avenue des Hauts-Fourneaux, 4362 Esch-sur-Alzette, Luxembourg), Martin N Rossor (Dementia Research Centre, Department of Neurodegenerative Disease, UCL Institute of Neurology, Queen Square, London, WC1N 3BG), Nick C Fox (Dementia Research Centre, Department of Neurodegenerative Disease, UCL Institute of Neurology Queen Square, London, WC1N 3BG), Jason D Warren (Dementia Research Centre, Department of Neurodegenerative Disease, UCL Institute of Neurology, Queen Square, London, WC1N 3BG), Maria Grazia Spillantini (University of Cambridge,

Department of Clinical Neurosciences, John Van Geest Brain Repair Centre, Forvie Site, Robinson way, Cambridge CB2 0PY), Huw R Morris (UCL, Department of Molecular Neuroscience, Russell Square House, 9-12 Russell Square House, London, WC1B 5EH), Patrizia Rizzu (German Center for Neurodegenerative Diseases-Tübingen, Otfried Muellerstrasse 23, Tuebingen 72076, Germany), Peter Heutink (German Center for Neurodegenerative Diseases-Tübingen, Otfried Muellerstrasse 23, Tuebingen 72076, Germany), Julie S Snowden (Institute of Brain, Behaviour and Mental Health, Faculty of Medical and Human Sciences, University of Manchester, Manchester, UK), Sara Rollinson (Institute of Brain, Behaviour and Mental Health, Faculty of Medical and Human Sciences, University of Manchester, Manchester, UK), Anna Richardson (Salford Royal Foundation Trust, Faculty of Medical and Human Sciences, University of Manchester, Manchester, UK), Alexander Gerhard (Institute of Brain, Behaviour and Mental Health, The University of Manchester, 27 Palatine Road, Withington, Manchester, M20 3LJ, UK), Amalia C Bruni (Regional Neurogenetic Centre, ASPCZ, Lamezia Terme, Italy), Raffaele Maletta (Regional Neurogenetic Centre, ASPCZ, Lamezia Terme, Italy), Francesca Frangipane (Regional Neurogenetic Centre, ASPCZ, Lamezia Terme, Italy), Chiara Cupidi (Regional Neurogenetic Centre, ASPCZ, Lamezia Terme, Italy), Livia Bernardi (Regional Neurogenetic Centre, ASPCZ, Lamezia Terme, Italy), Maria Anfossi (Regional Neurogenetic Centre, ASPCZ, Lamezia Terme, Italy), Maura Gallo (Regional Neurogenetic Centre, ASPCZ, Lamezia Terme, Italy), Maria Elena Conidi Regional Neurogenetic Centre, ASPCZ, Lamezia Terme, Italy), Nicoletta Smirne (Regional Neurogenetic Centre, ASPCZ, Lamezia Terme, Italy), Rosa Rademakers (Department of Neuroscience, Mayo Clinic Jacksonville, 4500 San Pablo Road, Jacksonville, FL 32224), Matt Baker (Department of Neuroscience, Mayo Clinic Jacksonville, 4500 San Pablo Road, Jacksonville, FL 32224), Dennis W Dickson (Department of Neuroscience, Mayo Clinic Jacksonville, 4500 San Pablo Road, Jacksonville, FL 32224), Neill R Graff-Radford (Department of Neurology, Mayo Clinic Jacksonville, 4500 San Pablo Road, Jacksonville, FL 32224), Ronald C Petersen (Department of Neurology, Mayo Clinic Rochester, 2001st street SW Rochester MN 5905), David Knopman (Department of Neurology, Mayo Clinic Rochester, 2001st street SW Rochester MN 5905), Keith A Josephs (Department of Neurology, Mayo Clinic Rochester, 2001st street SW Rochester MN 5905), Bradley F Boeve (Department of Neurology, Mayo Clinic Rochester, 2001st street SW Rochester MN 5905), Joseph E Parisi (Department of Pathology, Mayo Clinic Rochester, 2001st street SW Rochester MN 5905), William W Seeley (Department of Neurology, Box 1207, University of California, San Francisco, CA 94143, USA), Bruce L Miller (Memory and Aging Center, Department of Neurology, University of California, San Francisco, CA 94158, USA), Anna M Karydas (Memory and Aging Center, Department of Neurology, University of California, San Francisco, CA 94158, USA), Howard Rosen (Memory and Aging Center, Department of Neurology, University of California, San Francisco, CA 94158, USA), John C van Swieten (Department of Neurology, Erasmus Medical Centre, Rotterdam, The Netherlands; Department of Medical Genetics, VU university Medical Centre, Amsterdam, The Netherlands), Elise GP Dopper (Department of Neurology, Erasmus Medical Centre, Rotterdam, The Netherlands), Harro Seelaar (Department of Neurology, Erasmus Medical Centre, Rotterdam, The Netherlands), Yolande AL Pijnenburg (Alzheimer Centre and department of neurology, VU University medical centre, Amsterdam, The Netherlands), Philip Scheltens (Alzheimer Centre and department of neurology, VU University medical centre, Amsterdam, The Netherlands), Giancarlo Logroscino (Department of Basic Medical Sciences, Neurosciences and Sense Organs of the "Aldo Moro" University of Bari, Bari, Italy), Rosa Capozzo (Department of Basic Medical Sciences, Neurosciences and Sense Organs of the "Aldo Moro" University of Bari, Bari, Italy), Valeria Novelli (Medical Genetics Unit, Fondazione Policlinico Universitario A. Gemelli, Rome, Italy), Annibale A Puca (Cardiovascular Research Unit, IRCCS Multimedica, Milan, Italy; Department of Medicine and Surgery, University of Salerno, Baronissi (SA), Italy), Massimo Franceschi (Neurology Dept, IRCCS Multimedica, Milan, Italy), Alfredo Postiglione

(Department of Clinical Medicine and Surgery, University of Naples Federico II, Naples, Italy), Graziella Milan (Geriatric Center Frullone- ASL Napoli 1 Centro, Naples, Italy), Paolo Sorrentino (Geriatric Center Frullone- ASL Napoli 1 Centro, Naples, Italy), Mark Kristiansen (UCL Genomics, Institute of Child Health (ICH), UCL, London, UK), Huei-Hsin Chiang (Karolinska Institutet, Dept NVS, Alzheimer Research Center, Novum, SE-141 57, Stockholm, Sweden; Dept of Geriatric Medicine, Genetics Unit, M51, Karolinska University Hospital, SE-14186, Stockholm), Caroline Graff (Karolinska Institutet, Dept NVS, Alzheimer Research Center, Novum, SE-141 57, Stockholm, Sweden; Dept of Geriatric Medicine, Genetics Unit, M51, Karolinska University Hospital, SE-14186, Stockholm), Florence Pasquier (Univ Lille, Inserm 1171, DISTALZ, CHU 59000 Lille, France), Adeline Rollin (Univ Lille, Inserm 1171, DISTALZ, CHU 59000 Lille, France), Vincent Deramecourt (Univ Lille, Inserm 1171, DISTALZ, CHU 59000 Lille, France), Thibaud Lebouvier (Univ Lille, Inserm 1171, DISTALZ, CHU 59000 Lille, France), Dimitrios Kapogiannis (National Institute on Aging (NIA/NIH), 3001 S. Hanover St, NM 531, Baltimore, MD, 21230), Luigi Ferrucci (Clinical Research Branch, National Institute on Aging, Baltimore, MD, USA), Stuart Pickering-Brown (Institute of Brain, Behaviour and Mental Health, Faculty of Medical and Human Sciences, University of Manchester, Manchester, UK), Andrew B Singleton (Laboratory of Neurogenetics, National Institute on Aging, National Institutes of Health, Building 35, Room 1A215, 35 Convent Drive, Bethesda, MD 20892, USA), John Hardy (UCL, Department of Molecular Neuroscience, Russell Square House, 9-12 Russell Square House, London, WC1B 5EH), Parastoo Momeni (Laboratory of Neurogenetics, Department of Internal Medicine, Texas Tech University Health Science Center, 4th street, Lubbock, Texas 79430, USA)
